# Digital gene expression analysis of the zebra finch genome

**DOI:** 10.1186/1471-2164-11-219

**Published:** 2010-04-01

**Authors:** Robert Ekblom, Christopher N Balakrishnan, Terry Burke, Jon Slate

**Affiliations:** 1 Department of Animal and Plant Sciences, University of Sheffield, Alfred Denny Building, Western Bank, Sheffield S10 2TN, UK; 2 Department of Population Biology and Conservation Biology, Uppsala University, Norbyvägen 18 D, SE-75236 Uppsala, Sweden; 3 Institute for Genomic Biology and Department of Molecular & Cellular Biology, University of Illinois, 1206 West Gregory Drive MC-195, Urbana, IL 61801, USA

## Abstract

**Background:**

In order to understand patterns of adaptation and molecular evolution it is important to quantify both variation in gene expression and nucleotide sequence divergence. Gene expression profiling in non-model organisms has recently been facilitated by the advent of massively parallel sequencing technology. Here we investigate tissue specific gene expression patterns in the zebra finch (*Taeniopygia guttata*) with special emphasis on the genes of the major histocompatibility complex (MHC).

**Results:**

Almost 2 million 454-sequencing reads from cDNA of six different tissues were assembled and analysed. A total of 11,793 zebra finch transcripts were represented in this EST data, indicating a transcriptome coverage of about 65%. There was a positive correlation between the tissue specificity of gene expression and non-synonymous to synonymous nucleotide substitution ratio of genes, suggesting that genes with a specialised function are evolving at a higher rate (or with less constraint) than genes with a more general function. In line with this, there was also a negative correlation between overall expression levels and expression specificity of contigs. We found evidence for expression of 10 different genes related to the MHC. MHC genes showed relatively tissue specific expression levels and were in general primarily expressed in spleen. Several MHC genes, including MHC class I also showed expression in brain. Furthermore, for all genes with highest levels of expression in spleen there was an overrepresentation of several gene ontology terms related to immune function.

**Conclusions:**

Our study highlights the usefulness of next-generation sequence data for quantifying gene expression in the genome as a whole as well as in specific candidate genes. Overall, the data show predicted patterns of gene expression profiles and molecular evolution in the zebra finch genome. Expression of MHC genes in particular, corresponds well with expression patterns in other vertebrates.

## Background

Studies of molecular evolution have until recently focused on nucleotide divergence, while studies of variation in gene expression profiles have mainly been restricted to a few model species such as *Drosophila *and mice [1-4]. This is because the technologies for studying gene expression have not been available (or have been too costly to develop) for non-model species [5]. However, sequencing-based technologies for expression profiling can now be utilised to this end. By counting the number of reads generated by sequencing of cDNA from different genes in the transcriptome, one can get an estimate of the expression level of these genes in the particular tissues sampled [6]. A complementary approach is to scan publicly available databases of expressed sequence tags (ESTs) for the genes of interest. In addition to microarrays, these strategies, called digital transcriptomics, are today the most commonly used methods for investigating expression patterns [7]. Digital transcriptomics has received a great deal of attention, but the use of these methods has been restricted in many species by the requirement of having a reference genome to evaluate and analyse the data.

The advent of massively parallel (next-generation) sequencing is now starting to change this picture by providing a cost-effective way of generating large amount of sequence data in species where there is no prior knowledge of the genome sequence [8-10]. Next-generation sequencing technology generally generates millions of short sequence reads, each read being tens to hundreds of base pairs long, depending on the specific platform. This enables detection of genes even with very low expression levels. Roche 454-sequencing [11], in particular, generates reads that are long enough to be informative in the absence of a reference genome [12,13]. Here, we evaluate the use of 454-sequencing to investigate tissue specific gene expression profiles.

Next-generation sequencing can be used to not only describe genome-wide patterns of gene expression, but also to characterise specific gene families or genetic pathways. To illustrate this point, we use the ecologically important and widely studied genes of the major histocompatibility complex (MHC) for a more detailed analysis. These genes are a very common focus of studies that take a candidate gene approach to investigate functionally important genetic variation in immune function [14]. MHC genes are among the most variable of the vertebrate genomes [15-18]. In particular, the classical MHC genes (class I and class II) exhibit an extraordinary level of polymorphism. This polymorphism is strongly associated to the role of these genes in regulating and triggering the adaptive immune response. Studies have found links between nucleic acid variation in the MHC genes and resistance to parasites [19,20], sexually selected ornaments [21], mate choice [22], maternal-foetal incompatibilities [23] and local adaptation [24]. Typically studies of MHC variation have focused on sequence variation only in a few highly polymorphic regions of class I and class II genes, while variation in other genes, regions and expression levels has largely been ignored. The completion of the genome sequence of first the chicken (*Gallus gallus*) [25] and now of the zebra finch (Warren et al. in press) have opened the door for in-depth studies of organisation and expression of MHC genes in birds. There are striking differences in the way the adaptive immune defence operates in birds compared to mammals [26] and it could be envisioned that such studies will reveal new insights in the evolution of vertebrate immunity.

The aim of the present study was to investigate tissue-specific gene expression patterns in the zebra finch. With the sequencing of its genome, the zebra finch has taken a major step towards becoming an important model system for bird genomics [27,28]. Outside of some recent studies of gene expression in brain [29-31], however, little is known about genome-scale, and organism-wide patterns of gene expression in song birds. In this study we describe patterns of gene expression across six zebra finch tissues and explore the relationship between expression profiles of genes and characteristics of their molecular evolution. To this end, we use a next-generation sequencing (NGS) digital transcriptomics approach known as RNA-Seq [32,33]. This methodology was recently employed to study gene expression differentiation between two subspecies of crow (*Corvus corone*) [34], but as far as we are aware, this is the first time that a bird transcriptome has been characterised in multiple tissues using an NGS RNA-Seq approach. In addition to global patterns of gene expression, we highlight patterns of expression in the genes of the MHC. Because of the complex history of duplication among certain MHC genes, gene expression profiles have the potential to offer insight into the evolutionary fates of these duplicated genes. Importantly, characterizing the expression of MHC genes will also facilitate downstream studies of these genes in ecological contexts by identifying functionally important loci.

## Results

### Assembly of 454 sequencing reads

**Figure 1:**
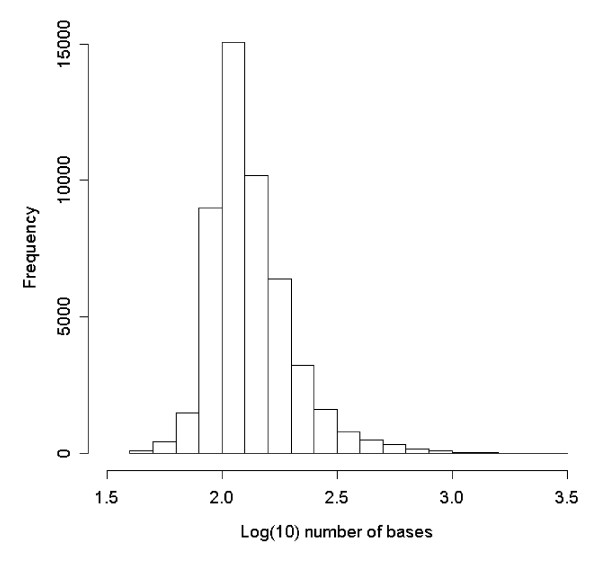
**Distribution of contig lengths (log) from 454 sequencing reads of all tissues combined**.

After trimming and removal of contaminant sequences a total of 1,882,439 reads were available, with a mean read length of 83 nucleotides. 741,917 of these reads were assembled (Additional file 1: Appendix s1) de-novo (the rest were kept as singletons) into 49,606 contigs with a mean contig length of 150 nucleotides (range 41-2,953; Figure 1) and a mean of 15 reads per contig. The total length of all contigs was 7,439 kb. For read and contig statistics for each tissue separately see Additional file 1: Appendix s2. 582 (1.2%) of the contigs showed signatures of multiple splice variants, as indicated by gaps in alignments between the contig and one or more of the reads that contribute to that contig.

### Expression levels for contigs

**Figure 2:**
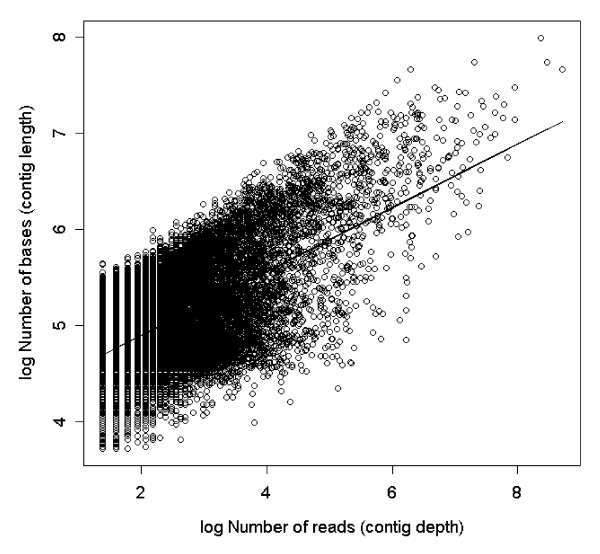
**Positive relationship between length (log number of bases) and depth (log number of reads) of contigs for the whole dataset (all tissues combined)**. The line represents a linear regression of the data (slope = 0.33, Intercept = 4.23, R^2 ^= 0.40, p < 0.0001).

**Figure 3:**
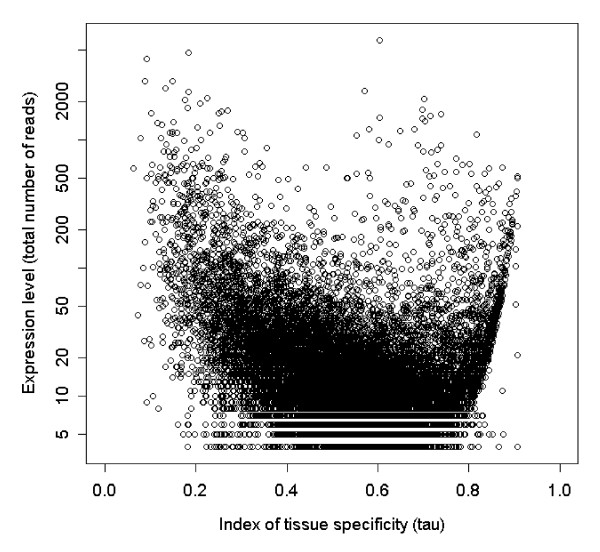
**Total expression level plotted against tissue specificity of gene expression (τ) for all contigs**.

Expression levels were highly variable between tissues and contigs. A vast majority of contigs were made up by only a few reads (median = 6) but some had indications of very high expression levels (maximum 6,028 reads; Additional file 1: Appendix s3). As would be expected (at least until a majority of the contigs are large enough to include the whole transcribed gene) there was a positive relationship between contig length (log number of base pairs) and contig depth (log number of reads; r = 0.636, df = 49,577, p < 0.0001, Figure 2, Additional file 1: Appendix s4). The tissue specificity of the expression (τ) of contigs was negatively correlated with the overall (log) amount of expression (r = -0.29, df = 49,076, p < 0.0001, Figure 3). This negative correlation could be the result of a sampling artefact during the calculation of τ. We found however, that the observed correlation was significantly (t_451 _= 8.35, p < 0.0001) stronger than the mean simulated correlation (mean r = -0.120) based on unbiased τ-values calculated from randomisations of re-sampled data. This suggests that there is indeed a sampling bias in the calculation of τ but that it is not strong enough to alone explain our observed correlation. Interestingly, τ seems to have a somewhat bimodal distribution (Additional file 1: Appendix s5) with peaks around 0.5 and 0.75, indicating that the genes may group into two different classes of tissue specificity. However, only a few contigs showed evidence of very low τ-values indicative of housekeeping genes.

### Outlier contigs with high expression levels

Three contigs were found to have strikingly high overall expression levels; following BLAST searches against the chicken and zebra finch gene databases they were found to represent Albumin (6,028 reads), Heat Shock 90 kDa Protein 1 Beta (4,305 reads) and NADH Dehydrogenase Subunit 1 (4,753 reads). All of these are considered to be so-called housekeeping genes (genes with equal expression across tissues and treatments) and are also highly expressed in mammals. Some contigs were conspicuous in having very strong expression in one or few tissues. Genes represented by these include Elongation Factor 1-Alpha (1,268 reads in embryo), Cytoplasmic Beta-Actin gene (1,185 reads in embryo), Haemoglobin Alpha (1,516 reads in spleen) and MHC Class II Associated Invariant Chain Ii (2,137 reads in spleen). One contig was found in high levels in testes (1,499 reads) but was almost completely absent in other tissues, the BLAST search revealed that this originates from a contamination with DNA from a freshwater planarian (*Schmidtea*). This is likely to have occurred in the laboratory that carried out the sequencing (the Washington University Genome Center), since the genome of *Schmidtea mediterranea *was being sequenced there at the same time as the zebra finch cDNA preparation. This contig, together with 26 other contigs resulting from contamination (mainly from planarians), was removed from the data before conducting downstream analyses.

### Coverage of the zebra finch transcriptome

13,562 contigs from the de-novo assembly and 118,165 of the non-assembled singletons gave significant BLAST hits against at least one predicted zebra finch gene. Since the contigs were generally much shorter than the total cDNA length of the gene it was commonly found that several different contigs matched the same gene. In total 11,793 zebra finch transcripts present in the BioMart database were found to correspond to the 454/EST transcriptome contigs and singletons. This represents 65% of the total characterised zebra finch transctiptome (18,241 unique transcripts). The transcripts are derived from 11,567 different genes, suggesting that more than one splice variant was detected (and placed in different contigs) for ~2% of the genes. We also identified potential novel splice variants for 270 of the expressed zebra finch genes, as indicated by gaps in the alignments of the contig and the gene prediction. On average 38% of the lengths of represented transcripts were covered by contig sequences and 370 transcripts were fully covered.

To further investigate the extent of transcriptomic coverage, we investigated the presence of known genes in various metabolic pathways and signalling cascades (Table 1)[35]. For the metabolic pathways about 85% of the genes were represented and for signalling cascades we found around 60%. 2,285 (19%) of all genes found were expressed in all investigated tissues and 2,998 (25%) were expressed exclusively in one tissue (Table 2). Out of the 36,044 contigs that did not give any matches to known predicted zebra finch transcripts, most (34,456) still gave highly significant BLAST hits (e < 1e-10) against the zebra finch genome sequence, suggesting that these represent transcribed regions that have not yet been annotated. The remaining 1,588 contigs (those that did not match either the annotated zebra finch genes or the genome sequence) may represent genes in regions of the genome that have not been sequenced and/or assembled in the current genome assembly, or additional contamination from other organisms that are not represented in GenBank.

**Table 1:** Number of genes for specific metabolic and signalling pathways identified in the zebra finch genome that were present in the transcriptome assembly presented here.

GO number	Biological process	Total # zebra finch genes	# present in this analysis	% represented	Mean τ (95% CI)
GO:0006096	Glycolysis	31	37	84	0.41 (0.30 - 0.51)
GO:0006094	Gluconeogenesis	7	6	86	0.56 (0.25 - 0.86)
GO:0006098	Pentose Phosphate	8	7	88	0.44 (0.31 - 0.56)
GO:0006101	Citrate metabolic processes	2	2	100	0.52 (NA)
GO:0007224	Hedgehog signalling pathways	15	7	47	0.50 (0.38 - 0.62)
GO:0007259	JAK/STAT cascade	8	5	63	0.55 (NA)
GO:0007219	Notch signalling	19	13	68	0.53 (0.42 - 0.65)
GO:0016055	WNT signalling	48	20	42	0.51 (0.38 - 0.64)
GO:0002224	Toll like receptor signalling	6	3	50	0.67 (NA)

-	MHC genes	16	10	62	0.60 (0.40 - 0.80)

Number of MHC related genes included in this study is also given. The mean index of tissue specificity of expression (τ) and its 95% CI (when more than two τ values) for each pathway is also given.

**Table 2:** Mean d_N_/d_S _(ω) values and index of tissue specificity of expression (τ) for genes with maximal expression in each of the investigated six tissues, together with 95% confidence intervals (CI).

Tissue	N_max _(N_unique_)	ω	95% CI (ω)	τ	95% CI (τ)
Embryo	2,033 (454)	0.132	0.120 - 0.144	0.438	0.430 - 0.446
Liver	1,347 (552)	0.157	0.150 - 0.164	0.518	0.506 - 0.530
Muscle	738 (348)	0.278	0.015 - 0.541	0.458	0.440 - 0.475
Skin	964 (427)	0.155	0.145 - 0.165	0.561	0.547 - 0.574
Spleen	1,000 (368)	0.161	0.140 - 0.182	0.492	0.479 - 0.505
Testes	2,996 (849)	0.165	0.148 - 0.182	0.542	0.534 - 0.549

N_max _represents the number of genes with maximal expression in each of the tissues and the number within brackets (N_unique_) is the number of genes which are expressed uniquely in that tissue.

### Analyses of gene expression profiles

**Figure 4:**
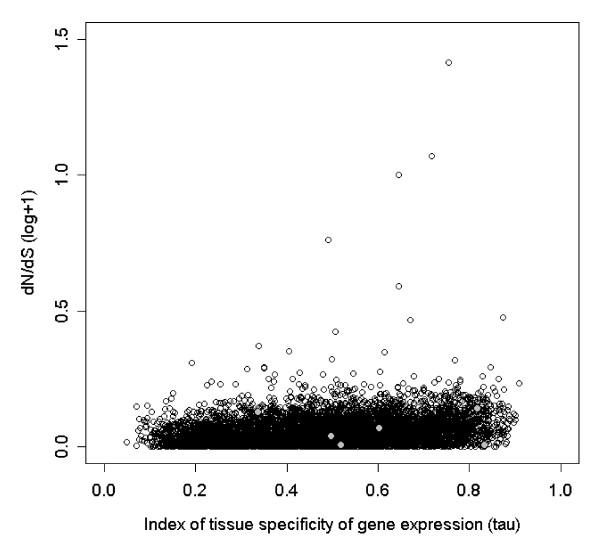
**Positive relationship between the rate of non-synonymous/synonymous substitution (log (ω+1)) and the index of specificity of gene expression (τ) for zebra finch against chicken comparisons of orthologous genes**. The grey data points represent MHC genes.

We found a positive correlation between tissue specificity of expression (τ) and the ratio of non-synonymous to synonymous substitution rate (ω) when compared to the chicken orthologue of the gene in question (r_s _= 0.20, df = 7,342, p < 0.0001, Figure 4). There was also a negative correlation between total expression level of the gene and ω (r_s _= -0.071, df = 10,711, p < 0.0001, Additional file 1: Appendix s6). There was a weak positive correlation between the length of the gene and the total level of gene expression (r_s _= 0.059, df = 10,711, p < 0.0001), and a negative correlation between gene length and τ (r_s _= -0.065, df = 7,342, p < 0.0001). There were differences in ω between the tissues in which genes were primarily expressed (Kruskal-Wallis test, χ^2 ^= 106.43, df = 5, p < 0.0001). Genes that were primarily expressed in the embryo had the lowest mean ω-value (Table 2). The expression specificity (τ) of genes also varied significantly between tissues of maximal expression (ANOVA, F_5 _= 87.5, p < 0.0001). The lowest tissue specificity was found in genes with primary expression in embryo and muscle, while the highest τ was found in genes with maximal expression in skin and testes (Table 2).

### Analysis of expression in relation to GO-terms

There were 20 gene ontology (GO) terms overrepresented (Fisher's adjusted p < 0.05) in genes with high levels of expression specificity (6 for "biological process", 2 for "cellular component" and 12 for "molecular function"; Additional file 1: Appendix s7). These represent processes such as cellular and organelle movement and specific enzymatic processes (for example "lipid metabolic processes" and "carboxypeptidase activity"). Some GO terms overrepresented in genes with high tissue specificity are associated with reproduction (such as "sperm motility") and immune defence (such as "foam cell differentiation", "serine-type endopeptidase activity" and "chemokine activity"). Genes identified as having low tissue specificity of gene expression were significantly overrepresented for 47 different GO terms (19 for "biological process", 14 for "cellular component" and 14 for "molecular function"; Additional file 1: Appendix s8). These terms generally represented functions such as protein synthesis and basal metabolic processes.

Gene ontology terms overrepresented in genes primarily expressed in embryo were mostly associated with cell division and protein synthesis (Additional file 1: Appendix s9). Gene ontology associated with genes with highest expression in liver indicated functions of specific metabolic processes - reactions involving oxygen and energy related processes (Additional file 1: Appendix s10). Also genes primarily expressed in muscles were associated with GO terms related to energy utilisation and especially the function of the mitochondria (Additional file 1: Appendix s11). In genes with the highest expression levels in skin there was an overrepresentation of GO terms related to cytoskeletal structures and cell proliferation (Additional file 1: Appendix s12). Of main interest in relation to MHC and immune function were genes with primary expression in spleen. GO terms associated with expression in this tissue include "leukocyte adhesion", "immune response", "cell surface receptor linked signal transduction" and "chemokine activity", but also several terms related to ribosomal activity (Additional file 1: Appendix s13). Lastly, there were a large number of GO terms overrepresented in genes with maximal expression in testes, including for example "spermatogenesis" and "microtubule motor activity" (Additional file 1: Appendix s14).

### Validation of expression profiling: "housekeeping" genes

We specifically investigated expression patterns in four widely used housekeeping genes that have been shown to have similar levels of expression over a wide range of tissues and treatments in birds [36,37]. Two highly expressed genes, Ubiquitin (UB) and Glyceraldehyde-3-Phosphate Dehydrogenase (GAPDH), were represented by 7,160 and 12,397 reads, respectively. The tissue specificities of gene expression (τ) for these were 0.15 for UB and 0.16 for GAPDH (both within the lower 3^rd ^percentile of the total distribution of τ) Two genes with medium expression levels also had low gene expression variation between tissues. Ribosomal Protein S13 (RPS13) was found in 395 reads and had a τ-value of 0.20, while 60S Ribosomal Protein L30 (RPL30) was found in 326 reads with a τ-value of 0.21 (within the lower 7^th ^percentile of the total distribution of τ).

### Case study of expression profiling: MHC genes

We surveyed expression data for 16 MHC-related genes found in the zebra finch assembly and targeted BAC sequencing (Balakrishnan et al. in review, GenBank: AC192433, AC191651, AC191861, AC192431, AC232985, AC232854). We found evidence for expression of ten different MHC related genes in the zebra finch (Table 3). Thus the coverage of these genes is comparable to the rest of the genome (Table 1). Among these there was evidence for one expressed MHC class I loci but we did not find expression of any MHC class II loci in the present dataset. This is not to say that there are no expressed MHC class II molecules in the zebra finch, but only that these genes are expressed at too low levels in the sampled tissues to be detected using our methodology. The expression patterns of MHC genes were generally tissue specific (τ ranging from 0.336 to 0.833), with the highest expression levels for most genes in spleen. A detailed presentation of the expression for specific MHC genes can be found in a separate supplementary text (Additional files 2 and 3). One of the MHC genes, CD74 (Ii) presents a case of alternative splicing. As is true in many other species, we found evidence for at least two differently spliced isoforms of this gene, represented by different contigs in our 454 sequence assembly (Additional file 2; Figure s3).

**Table 3:** Expression of zebra finch MHC genes in seven different tissues expressed as number of transcripts per million (TPM) [57].

Gene	Brain, EST	Embryo	Liver	Muscle	Skin	Spleen	Testes	τ
TUBB	21.7	3.1	0.0	0.0	0.0	0.0	0.0	-
TRIM7.2	97.8	0.0	0.0	3.1	0.0	0.0	0.0	0.833
TRIM39	0.0	0.0	2.5	0.0	4.0	6.9	3.3	0.519
TRIM27	10.9	3.1	0.0	0.0	0.0	0.0	0.0	-
Ii	76.1	182.2	323.2	887.5	241.7	4,060.4	250.2	0.336
Class I	130.4	6.2	106.9	18.4	31.7	1,695.0	110.1	0.497
CIITA	0.0	0.0	0.0	0.0	0.0	10.4	0.0	-
CD1A	0.0	0.0	2.5	0.0	0.0	55.6	0.0	0.817
BRD2	21.7	0.0	0.0	0.0	0.0	0.0	0.0	-
B2M	10.9	0.0	45.8	9.2	27.7	896.1	43.4	0.602

Library size	92,040	323,897	392,890	325,646	252,349	287,902	299,755	

The total number of reads in each tissue library after trimming (library size) and the tissue specificity of gene expression (τ) are also given (τ values based on three or fewer reads are omitted, see Methods).

## Discussion

We have used transcriptomic data from six different tissues, generated by 454-sequencing [11], to investigate expression patterns of different zebra finch genes. Our results highlight, in a new evolutionary lineage, a number of trends in the evolution of gene expression profiles. Genes with a high degree of tissue specificity in expression levels also had high non-synonymous to synonymous rate of nucleotide substitutions (d_N_/d_S_), while genes with high overall expression levels had low d_N_/d_S _ratios. Thus genes with a more specialised function (lower overall expression and higher degree of tissue specificity) seem to be evolving at a higher rate (or with less constraint) than genes with a more general function (high overall expression and low degree of tissue specificity). These results recapitulate those of Axelsson and co-workers [38] who analysed chicken expression profiles in conjunction with sequence divergence data from chicken and zebra finch. Similar patterns of molecular evolution and expression specificity have also been found in mammals [39]. One important consequence of this finding for future studies of gene expression is that genes under strong positive selection might be missed if RNA from the appropriate tissues is not sequenced. In other words, the genes that are likely to be relevant for explaining genetic variation in ecologically important processes such as host-parasite co-evolution or reproduction [40] may be relatively less likely to be sequenced. This is particularly relevant in the present study system because the vast majority of gene expression studies in passerine birds have focussed on a single tissue, the brain.

Overall about 65% of the annotated zebra finch transcripts were covered by 454 sequencing in this study. An analysis of genes in well characterised metabolic pathways and signalling cascades [35] also corroborate this number. There is also some indication that more than one splice variant [41] was detected for some of the genes. Most of the contigs that did not match any of the annotated zebra finch transcripts still gave highly relevant hits against the zebra finch genome, suggesting that these represent novel genes that have yet to be annotated in the zebra finch genome. A few contigs that did not match anywhere in the zebra finch genome could either be part of genetic regions that have not been sequenced in the present zebra finch genome assembly or may represent contamination from other organisms. Higher coverage transcriptome sequencing will be needed to complete the zebra finch transcriptome and to fully characterize splice-variants.

Genes primarily expressed in embryo had low d_N_/d_S _ratios, while genes with the highest expression in testes showed high ratio. Low d_N_/d_S _ratios of embryonically expressed genes may represent stabilizing selection and high evolutionary constraint on core developmental and housekeeping genes [42]. High d_N_/d_S _in testes-expressed and reproductive genes has also been observed in human versus chimpanzee comparisons [40], in *Drosophila *[43,44] and in mice [45]. Such a pattern may be attributable to sexual selection acting on genes important for traits involved in reproduction. High d_N_/d_S _values of genes expressed primarily in spleen is also concordant with previous studies showing high rate of evolution in genes involved in the immune system [46]. Several of the MHC genes investigated in this study had primary expression in spleen and high d_N_/d_S _ratios of these genes are often seen as an indication of balancing selection acting on them [47].

In addition to performing genome wide analyses we also used the 454 transcriptome sequence data to investigate specific genes of interest. In particular, special attention was given to genes of the major histocompatibillity complex (see Additional file 2 for details). We found evidence for expression of ten different genes associated with the major histocompatibility complex. Most of these were primarily expressed in spleen, although there were also high levels of expression in brain and liver for some. Expression in the spleen is hardly surprising given the function of spleen in the immune defence. The expression of MHC genes in the brain however, was only relatively recently discovered in mammals [48] and has not been previously described in birds. It will be of interest to determine whether the role of the MHC in the brain is conserved across vertebrates. Furthermore, several gene ontology terms related to immune response were overrepresented in genes with primary expression in spleen. Some GO terms related to immune response were also overrepresented in genes with high tissue specificity, indicating that many immune genes are expressed mainly in a few specialised tissues.

For a few known MHC genes we could not detect any expression. This illustrates the fact that one may not necessarily find specific genes of interest in a next generation transcriptome sequencing dataset, especially if they are expressed at very low levels or only in specific tissues or life history stages. On the other hand, ongoing development of next generation sequencing technologies means that deeper coverage will be obtained enabling gene finding of lowly expressed genes. Coverage of MHC genes was within the range of other well characterised groups of genes related to specific metabolic and signalling pathways. These genes had medium levels of tissue specificity of expression, and there was a tendency for MHC genes to have higher levels of expression specificity (Table 1).

In expression profiling it is preferable to use sequences from a non-normalized cDNA library to avoid bias in the estimates of expression individual genes [49]. In our case the only data available for gene expression in different tissues came from cDNA libraries that were normalized to increase the abundance of rare transcripts [50]. Thus there is a risk that our expression estimates might be biased. In particular the expression levels of rare transcripts are probably overestimated while the levels for very common transcripts should be underestimated. This also means that estimates of tissue specificity of gene expression (τ) may be underestimated for individual genes. We argue, however, that the comparative analyses presented here can be performed using this dataset. There are at least four lines of evidence that these analyses are valid. 1) There is still considerable variation in expression levels between the different genes and tissues in our study, with many genes only expressed in one or a few tissues. 2) The analysis concerning gene expression gave results in the predicted direction. For example there was a positive relationship between specificity of gene expression and d_N_/d_S _ratio [38]. 3) The expression of most MHC genes was by far strongest in spleen which is what would be predicted for genes involved in immune defence. Further, GO terms overrepresented for genes with maximal expression in a certain tissue seemed to correspond well to those expected given the biological functions of the different tissues. 4) The expression levels of several housekeeping genes seemed to be stable across the different tissues analysed here.

One potential explanation for our failure to find 6 of the 16 MHC genes surveyed is that the relatively short contigs generated here, in combination with oligo dT priming, produced a strong 3' bias in the 454 sequencing. Indeed, many of the 454 reads fell in the 3' untranslated region (UTR) of genes (Additional file 2: Figure s4). It is therefore possible that these MHC genes were expressed, but the sequence reads only included UTR sequence. To investigate this issue we collected information from the avian MHC genes where the 3' UTR has been sequenced. UTR regions of avian MHC genes are not well-described at this point but we found 3' UTR sequence data for MHC class IIB from chicken, turkey, quail, New Zealand robin (*Petroica australis*), Bengalese finch (*Lonchura striata*) and zebra finch (locus 2 from the genome sequence). For MHC class I we found data from chicken, turkey, quail, mallard duck (*Anas platyrhynchos*) and great reed warbler (*Acrocephalus arundinaceus*), and we also included data from duck CD74 (Ii). These sequences were blasted against all zebra finch 454 contigs and positive matches were verified by a reciprocal BLAST against the zebra finch genome and chicken transcriptome databases. Only two of our contigs matched the 3' UTR MHC sequences, both representing the CD74 (Ii) transcript. Therefore it is unlikely that the failure to detect more MHC genes can be attributed solely to the short, and 3' UTR biased, contigs we assembled. New and improved methods for library preparation are now used to deal with this problem of 3' bias.

In general, the contigs produced using de-novo assembly of the 454-reads only partially covered the gene transcripts, with a mean contig length of only 150 nucleotides. These data were produced using the first generation of the 454-sequencing system (GS20) for which maximal read lengths were only around 125 bp. With application of the new generation of 454-sequencing (GS FLX Titanium), which generates more and longer reads, one would expect to get longer contigs and more contigs covering the whole of the gene coding sequence [51]. On the other hand deeper coverage of the transcriptome, and expression data on more genes, would be obtained using Illumina/Solexa or ABI SOLLiD technology. Both of these approaches generates a much larger amount of reads compared to 454 sequencing but at a cost of much shorter reads. They are thus particularly useful for species, like the zebra finch, that have a characterized genome sequence.

This study highlights the utility of next-generation sequencing data for expression pattern profiling. The zebra finch genome sequence was recently released and this, together with the gene predictions available, has been very useful when analysing the data. Still, this methodology would also work well when addressing a non-model species without any prior genome information [10]. In particular, the long read lengths of the new Titanium 454-generation means that many expressed genes can be identified using comparative sequence analysis against genomes of distantly related species. The combination of data on sequence and gene expression variation makes this strategy useful for future studies in novel species. However, our study also shows that it may not always be possible to find and sequence specific genes of interest using whole-transcriptome sequencing. For example, we did not find any MHC class II, TAP or tapasin sequences, even though there is no reason to believe that these are not present and expressed in the zebra finch genome. It may be that gene capture methods [52] or more efficient cDNA normalization and random primed libraries are needed to be able to pick up specific and very rare transcripts. Another approach to improving the discovery of genes specifically involved in the immune system would be to boost an immune response prior to cDNA sampling.

## Conclusions

Our analysis of the zebra finch transcriptome extends conserved patterns of gene expression profiles and molecular evolution to the avian lineage. Genes with low overall and tissue specific expression were shown to evolve at a higher rate than genes with high and unspecific expression levels. Such genes were also shown to be related to biological functions such as reproduction and immune response. Furthermore genes with primary expression in spleen were often related to the immune function (for example several MHC genes). Our results highlight the usefulness of next-generation sequence data for investigating expression profiles in the genome as well as in specific candidate genes. However, as illustrated by our survey of MHC genes, it is far from certain that all genes of interest will be present in a given transcriptome sequencing run. Therefore care must thus be taken to ensure sampling of the appropriate tissues and life stages if the aim of the sequencing run is to examine specific gene families or physiological pathways.

## Methods

### Sequence data

Gene expression was analysed using 454 pyrosequencing data generated by sequencing of cDNA from six different tissues (Embryo, Liver, Muscle, Skin, Spleen and Testes) of from pooled samples from six different zebra finches in the University of Sheffield colony [53]. Raw data (.sff files) from the GS20 sequencer were kindly provided by Wesley C. Warren (The Genome Center, Washington University School of Medicine). This represent two sequencing runs of cDNA from each tissue type, totalling 1,961,888 reads. Library construction of polyadenylated cDNA was performed using a variation of the Clontech SMART system, in which the 5' and 3' PCR adapters contain type IIs restriction enzyme sites (*Mme*I). The optimally-cycled product was then normalized using a duplex-specific nuclease (DSN) that preferentially digests double-stranded DNA in the presence of single-stranded DNA (Trimmer; Evrogen). For more details about cDNA synthesis and normalization see [50]. The produced sequence reads are also available as fasta files in the NCBI trace archive http://www.ncbi.nlm.nih.gov/Traces/trace.cgi?cmd=retrieve&s=search&m=obtain&retrieve=Search&val=SPECIES_CODE%3D'TAENIOPYGIA+GUTTATA'+AND+CENTER_NAME%3D'WUGSC'+AND+TRACE_TYPE_CODE%3D'454'. For expression analysis of MHC genes we also used EST libraries from zebra finch brain tissue downloaded from the NCBI website. Coding sequences from manually-annotated MHC genes were obtained by BLAST searches and HMMER gene prediction of the zebra finch genome, as described in Balakrishnan et al. (in review). After screening zebra finch BAC libraries using probes designed for MHC genes, seven BAC clones were sequenced at 6x coverage (Balakrishnan et al. in review). Predicted zebra finch gene sequences (cDNA, version 3.2.4.54) and chicken protein sequences (version 2.52) were downloaded from the ENSEMBL ftp site http://www.ensembl.org/info/data/ftp/index.html.

### 454 assembly

Trimming and assemblies (both de-novo and templated, see below) of 454 sequence fragments were performed using SeqMan NGen version 2.0 (DNASTAR, Inc.). The sequences were trimmed of low-quality sequence, poly-A tails, Smart primer sequence from cDNA synthesis and 454 adaptor sequence before assembling into contigs. In order to avoid falsely joining reads that do not belong to the same gene, we increased the match size to 41 base pairs. This parameter defines the length of sequences common to two or more sequences that are used to join reads together into contigs. For other parameters we used default values or values suggested in the software manual for assembling 454 data (for complete trimming and assembly parameters see Additional file 1: Appendix s1). 454 reads for all six tissues were first combined in a full data de-novo assembly. In order to identify contigs with multiple splice variants we also searched for gaps (defined here as more than 15 bases long) in the alignments between all the individual reads and the best matching contig. Alternative isoforms would be expected to generate alignment gaps if a contig contains an extra (or different) exon which is not present in the read. In order to check for tissue specificity of expression, another assembly was then made for each tissue separately using the contigs created by the full data assembly as a sequence template. To investigate expression of MHC genes specifically we also performed a templated assembly using zebra finch chromosome 16 and MHC containing BAC sequences as a reference sequence (for more details about the MHC analyses see Additional file 2).

### Transcriptomic analysis

All of the contigs and singletons from the de-novo assembly of 454 reads from all six different tissues were blasted (BLASTN) against the Ensembl zebra finch gene predictions using a cut-off e-value of 1e-10. Only the best BLAST (minimum e-value, maximum length) hit from each contig was extracted. For each unique gene we then combined the data on number of reads for each corresponding contig and singletons (since most contigs did not cover the whole gene it was common that several different contigs and singletons gave BLAST hits to different parts of the same gene). To calculate the proportion of the individual genes that were covered with our transcripts we used the length of the gene divided by the sum of the length of all contigs aligned to that gene. In the few cases where the total contig length was larger than the gene length (probably due to overlapping contigs) the gene coverage was set to 100%. We also searched for gaps in the alignments between the contigs and the Ensembl gene predictions, as these are indications of the presence of novel splice variants in the expression data. Data on gene length, name, genomic location and d_N_/d_S _ratio (compared to the chicken orthologue) were then extracted from BioMart http://www.ensembl.org/biomart/martview/. Values of d_N_/d_S _for MHC genes not annotated in Ensembl were calculated using the codeml model in PAML4 [54] using the IDEA interface [55]. To investigate transcriptome coverage of our contigs and reads matching Ensembl contigs we searched specifically for genes in well characterised metabolic pathways and signalling cascades. The specific pathways investigated were chosen based on similar studies e.g. [35]. We also searched (BLASTN) the current assembly zebra finch genome (version 3.2.4) for matches to all contigs that did not produce good hits to any annotated gene models in order to identify candidates for new and non-annotated zebra finch genes.

### Tissue Specificity of Gene Expression

We calculated the index of tissue specificity of gene expression (τ)[56], using the guidelines in [57]. Thus, the number of transcripts per million (TPM) was set to 2 for tissues with no detected expression of the gene in question. Furthermore τ estimates based on 3 or fewer reads were removed from the analyses. This was done to reduce the effect of sampling stochasticity when expression levels were very low. The theoretical range of τ for a specific gene varies between 0 and 1, where 0 means that the gene is equally expressed in all studied tissues (housekeeping genes) and values approaching 1 means that the gene is expressed specifically in one tissue [56]. The tissue of maximal expression was defined as the tissue with the highest number of reads for a specific gene. Genes with less than four reads were also excluded from lists of maximal expression.

### Simulation to investigate bias in τ

To investigate possible bias in the calculation of τ, we also performed a simulation of τ calculated from re-sampled data. For each of the 452 levels of gene expression in our data we randomly drew the same number of contigs as observed from the full distribution of expression levels while keeping the relative expression levels between tissues constant. This procedure was iterated enough times to get the same number of data points as for the observed data. As these data points all come from contigs with the same expression level, τ values calculated from these should be unbiased with respect to expression. We then calculated the correlation coefficient between total gene expression and τ for each of these 452 simulated datasets and compared these to the observed correlation coefficient for the original dataset.

### Gene ontology analysis

The five hundred genes with the highest and the five hundred genes with lowest tissue specificity of expression, as well as all genes with maximal expression for each of the six tissues, were compared against all other zebra finch genes with respect to associated gene ontology (GO) terms. GO terms more common in these genes than expected by chance (adjusted Fishers p < 0.05) were identified using the CORNA algorithm [58], applied using the web interface provided by Michael Watson at the Institute for Animal Health http://bioinformatics.iah.ac.uk/tools/GOfinch.

### Statistical analyses

Sequence similarity searches were performed using a stand-alone version of the BLAST (2.2.18) package [59]. Handling of BLAST output files, assembly results and statistical analyses were performed in R (2.7.2) statistical computing language [60]. Total expression levels and d_N_/d_S _ratios of genes were not normally distributed (Kolmogorov-Smirnov test, p < 0.0001) and therefore non-parametric tests were used for analyses involving these.

## Authors' contributions

RE conducted the analyses and prepared the manuscript. CNB prepared and provided BAC sequences and analyses of these and also contributed to the writing of the manuscript. TB and JS helped plan the work, provided significant feedback on the results and the manuscript. All authors have read and approved the final version of the manuscript.

## Supplementary Material

Additional file 1 **Appendix s1 - s14**. Additional tables and figures Click here for file

Additional file 2 **Appendix s15**. Detailed survey of MHC genes Click here for file

Additional file 3 **Appendix s16**. Alignment of the zebra finch MHC class I gene Click here for file
