# Supplementary material for: Digital gene expression analysis of the zebra finch genome
Source: BMC Genomics. 2010 Apr 1;11:219. doi: 10.1186/1471-2164-11-219 (PMC2996964; doi:10.1186/1471-2164-11-219)
Supplement: Additional file 1 — Appendix s1 - s14. Additional tables and figures [file 1471-2164-11-219-S1.PDF]

Appendix s1. Parameters used in NGen 2.0 for trimming and assembly of 454 reads.

Quality trimming parameters

- Window length: 30
- Average quality cut off: 14

Poly A trimming parameters

- Mer length: 5
- Minimum number of matching mers: 8

Vector trimming parameters (smart primers and 454 adaptor sequences)

- Mer length: 8
- Minimum number of matching mers: 3
- Minimum trim length: 15

Assembly parameters

- Use repeat handling: false
- Match size: 41
- Minimum match percentage: 90
- Match spacing: 10
- Minimum sequences in contig: 4 (de-novo assembly only)
- Gap penalty: 30
- Maximum number of gaps per 1000 bp: 15

Appendix s2. Details of assembl yresults for each tissue.

	Total	Embryo	Liver	Muscle	Skin	Spleen	Testes
Number of contigs	49,606	28,855	22,873	17,326	25,384	26,595	25,444
Number of reads in contigs	741,917	151,080	143,539	130,380	114,308	134,826	170,417
Mean number of reads per contig	15.0	3.0	2.9	2.6	2.3	2.7	3.4
Number of singletons	1,140,522	172,817	249,351	195,266	138,041	153,076	129,338
Number of trimmed (removed) reads	79,449	15,327	10,284	7,745	10,106	19,629	16,358

Appendix s3. Distribution of contig depth (number of reads per contig) for the complete dataset (all tissues combined).

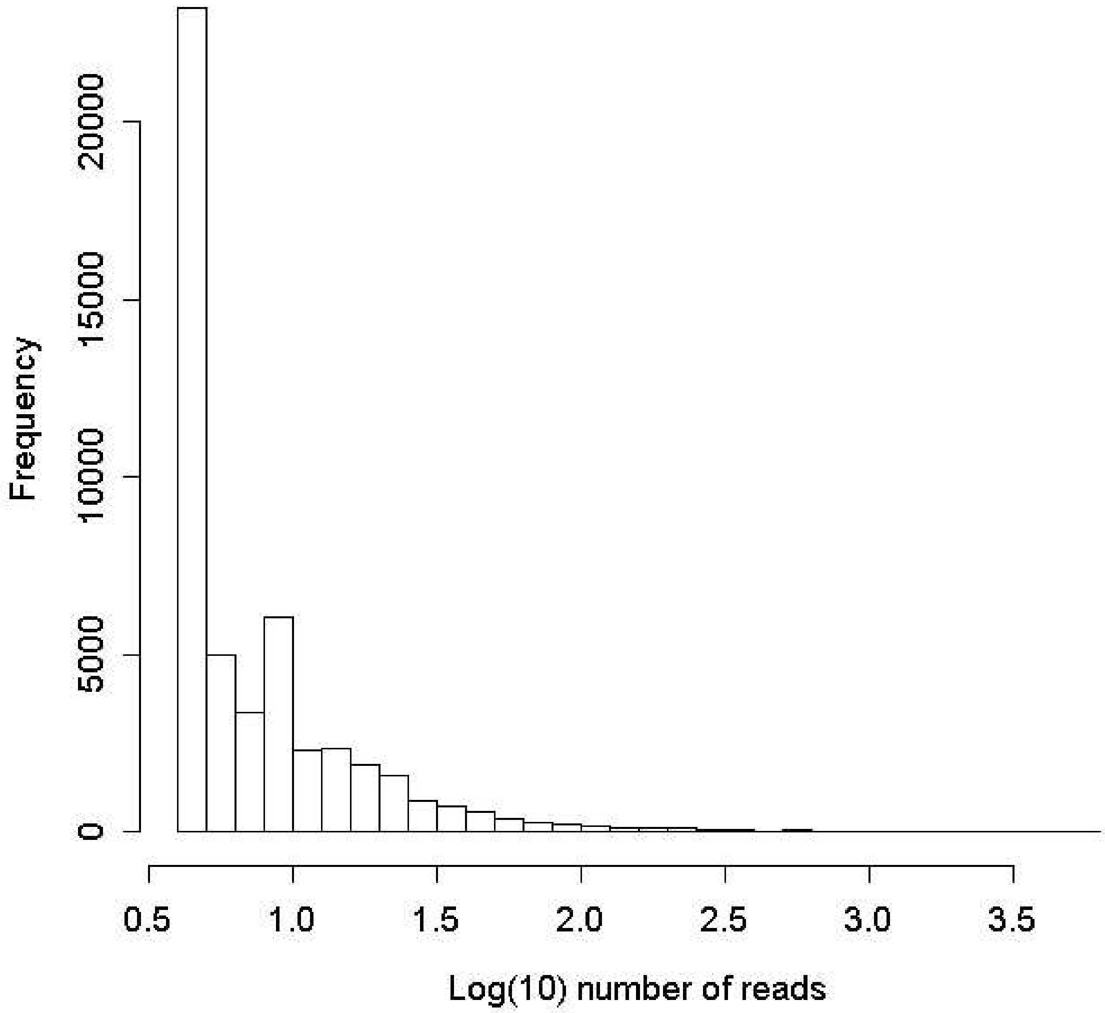

Appendix s4. Contig depth (log number of bases) against length (log number or reads) for each tissue separately.

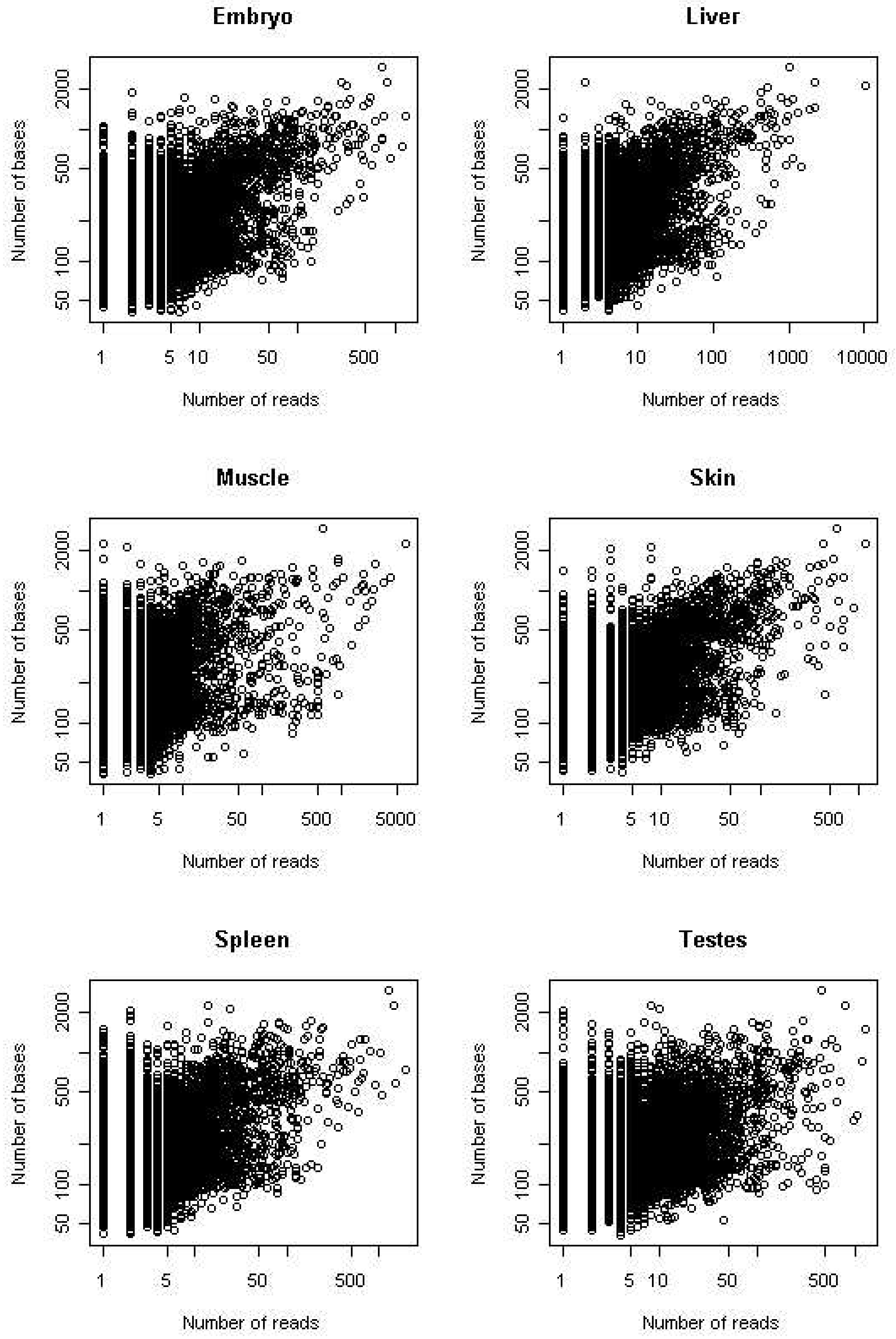

Appendix s5. Distribution of τ (tissue specificity of expression) for all contigs.

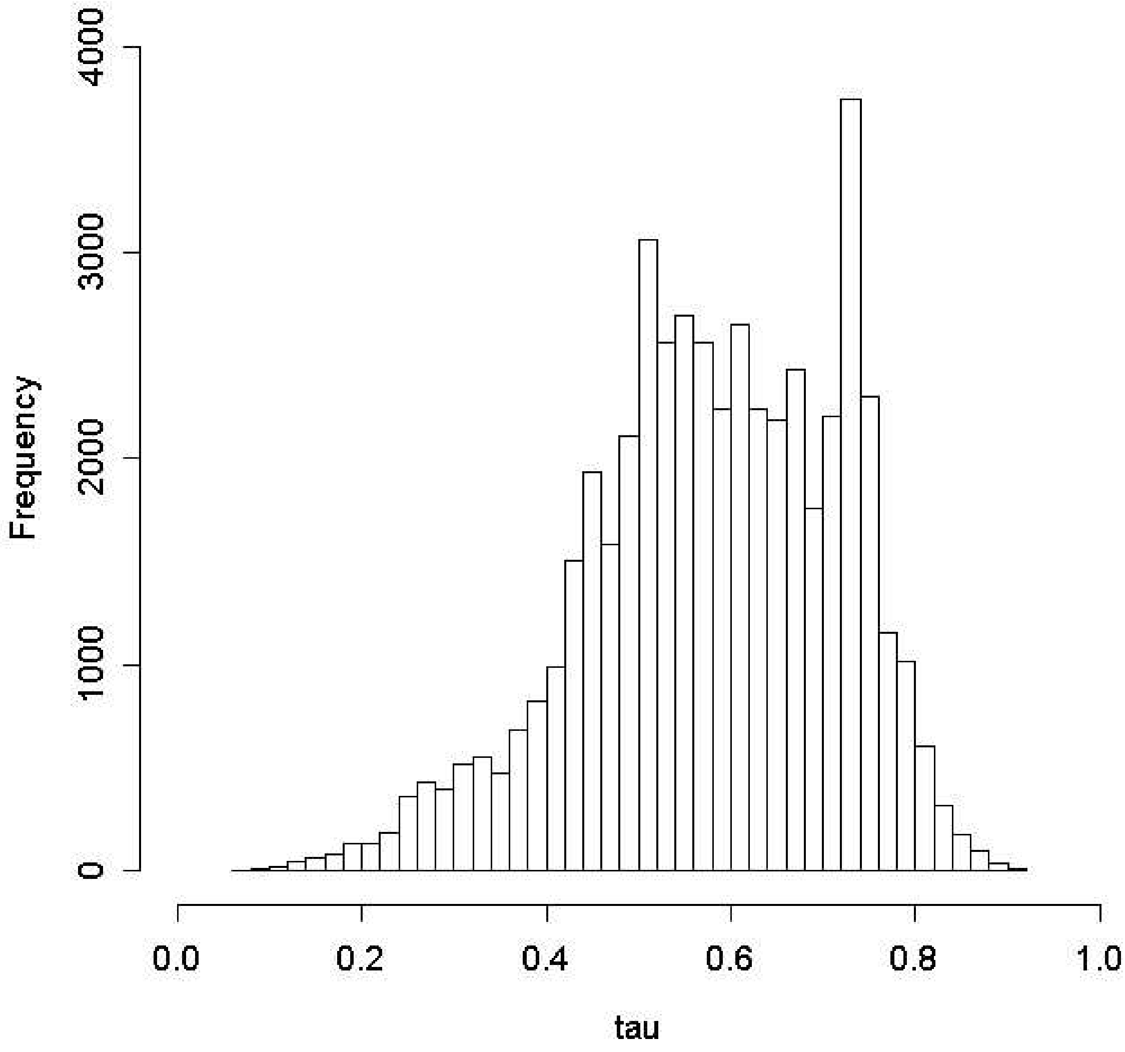

Appendix s6. **Non-synonymous to synonymous substitution rate (log ω+1) plotted against total level of gene expression (log number of reads across all tissues). Grey points represent MHC genes.**

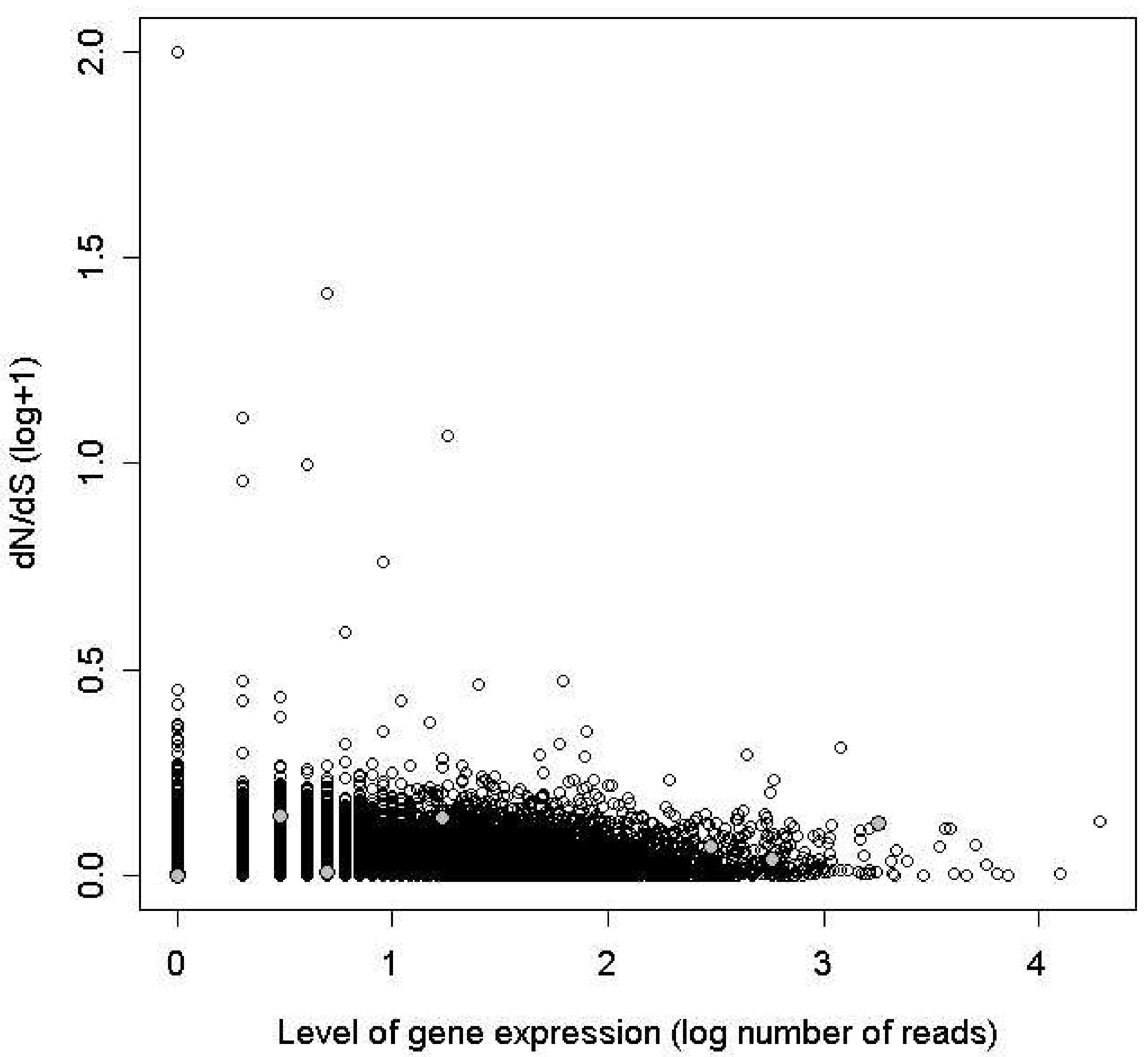

Appendix s7. Gene ontology terms that are overrepresented in genes with high tissue specificity of gene expression (τ). Terms with significant effects (Fisher's adjusted p < 0.05) are reported.

GO	go_description	total	expectation	observation	p
biological process					
GO:0006508	proteolysis	395	12	30	0.00032
GO:0007018	microtubule-based movement	76	2	9	0.026
GO:0030317	sperm motility	7	0	3	0.032
GO:0010744	positive regulation of foam cell differentiation	2	0	2	0.032
GO:0006559	L-phenylalanine catabolic process	2	0	2	0.032
GO:0006629	lipid metabolic process	103	3	10	0.032
cellular component					
GO:0005874	microtubule	33	1	7	0.0045
GO:0005882	intermediate filament	82	2	10	0.012
molecular function					
GO:0004252	serine-type endopeptidase activity	123	4	19	2.00E-06
GO:0004867	serine-type endopeptidase inhibitor activity	71	2	14	5.00E-06
GO:0003824	catalytic activity	648	19	39	0.0027
GO:0005506	iron ion binding	138	4	13	0.016
GO:0003777	microtubule motor activity	65	2	8	0.032
GO:0004574	oligo-1,6-glucosidase activity	2	0	2	0.032
GO:0004575	sucrose alpha-glucosidase activity	2	0	2	0.032
GO:0004397	histidine ammonia-lyase activity	2	0	2	0.032
GO:0016211	ammonia ligase activity	2	0	2	0.032
GO:0004180	carboxypeptidase activity	8	0	3	0.042
GO:0005200	structural constituent of cytoskeleton	93	3	9	0.047
GO:0008009	chemokine activity	18	1	4	0.047

Appendix s8. Gene ontology terms that are overrepresented in genes with low tissue specificity of gene expression (τ). Terms with significant effects (Fisher's adjusted p < 0.05) are reported.

GO	go_description	total	expectation	observation	p
biological process					
GO:0006412	translation	206	7	43	1.40E-19
GO:0006457	protein folding	75	3	15	4.20E-06
GO:0044267	cellular protein metabolic process	17	1	7	6.30E-05
GO:0006122	mitochondrial electron transport, ubiquinol to cytochrome c	7	0	4	0.0024
GO:0006413	translational initiation	24	1	6	0.0048
GO:0006886	intracellular protein transport	254	9	21	0.0094
GO:0006511	ubiquitin-dependent protein catabolic process	109	4	12	0.01
GO:0006096	glycolysis	32	1	6	0.016
GO:0044262	cellular carbohydrate metabolic process	6	0	3	0.016
GO:0006302	double-strand break repair	14	0	4	0.02
GO:0015031	protein transport	193	7	16	0.02
GO:0019642	anaerobic glycolysis	2	0	2	0.021
GO:0007031	peroxisome organization	7	0	3	0.021
GO:0006415	translational termination	7	0	3	0.021
GO:0006414	translational elongation	7	0	3	0.021
GO:0006464	protein modification process	93	3	10	0.022
GO:0015986	ATP synthesis coupled proton transport	51	2	7	0.027
GO:0030163	protein catabolic process	18	1	4	0.042
GO:0000398	nuclear mRNA splicing, via spliceosome	9	0	3	0.042
cellular component					
GO:0005840	ribosome	148	5	38	2.90E-20
GO:0005739	mitochondrion	382	13	41	1.90E-08
GO:0005839	proteasome core complex	14	0	7	1.50E-05
GO:0005829	cytosol	149	5	20	1.70E-05
GO:0043234	protein complex	50	2	10	0.00039
GO:0005832	chaperonin-containing T-complex	4	0	3	0.0054
GO:0030117	membrane coat	28	1	6	0.0094
GO:0022627	cytosolic small ribosomal subunit	11	0	4	0.01
GO:0005743	mitochondrial inner membrane	134	5	13	0.016
GO:0000276	mitochondrial proton-transporting ATP synthase complex, coupling factor F(o)	6	0	3	0.016
GO:0005783	endoplasmic reticulum	170	6	15	0.017
GO:0005768	endosome	23	1	5	0.019
GO:0005788	endoplasmic reticulum lumen	8	0	3	0.031
GO:0005746	mitochondrial respiratory chain	3	0	2	0.048
molecular function					
GO:0003735	structural constituent of ribosome	140	5	37	2.90E-20
GO:0051082	unfolded protein binding	62	2	14	2.80E-06
GO:0004298	threonine-type endopeptidase activity	14	0	7	1.50E-05
GO:0003743	translation initiation factor activity	29	1	8	0.00031
GO:0004175	endopeptidase activity	12	0	5	0.0017
GO:0005515	protein binding	3375	117	154	0.0032
GO:0003697	single-stranded DNA binding	14	0	5	0.0032
GO:0004129	cytochrome-c oxidase activity	14	0	5	0.0032
GO:0008121	ubiquinol-cytochrome-c reductase activity	8	0	4	0.0036
GO:0000166	nucleotide binding	303	10	24	0.0054
GO:0008565	protein transporter activity	47	2	8	0.006
GO:0003747	translation release factor activity	6	0	3	0.016
GO:0004459	L-lactate dehydrogenase activity	2	0	2	0.021
GO:0015078	hydrogen ion transmembrane transporter activity	26	1	5	0.027

Appendix s9. Gene ontology terms that are overrepresented in genes that are primarily expressed in embryo. Terms with significant effects (Fisher's adjusted p < 0.05) are reported.

GO	go_description	total	expectation	observation	p
biological process					
GO:0006412	translation	206	27	57	8.60E-06
GO:0006418	tRNA aminoacylation for protein translation	44	6	17	0.0037
GO:0006396	RNA processing	52	7	18	0.0092
GO:0007067	mitosis	14	2	8	0.018
cellular component					
GO:0005737	cytoplasm	1077	139	191	0.00085
GO:0005840	ribosome	148	19	40	0.0012
GO:0005634	nucleus	2161	279	341	0.0037
GO:0005664	nuclear origin of replication recognition complex	4	1	4	0.034
GO:0005654	nucleoplasm	20	3	9	0.045
molecular function					
GO:0005515	protein binding	3375	435	558	1.50E-09
GO:0003723	RNA binding	209	27	61	4.60E-07
GO:0003682	chromatin binding	74	10	28	2.80E-05
GO:0004812	aminoacyl-tRNA ligase activity	47	6	18	0.003
GO:0003735	structural constituent of ribosome	140	18	37	0.0037
GO:0004386	helicase activity	105	14	28	0.026
GO:0005003	ephrin receptor activity	12	2	7	0.034
GO:0003755	peptidyl-prolyl cis-trans isomerase activity	19	2	9	0.034

Appendix s10. Gene ontology terms that are overrepresented in genes that are primarily expressed in liver. Terms with significant effects (Fisher's adjusted p < 0.05) are reported.

GO	go_description	total	expectation	observation	p
biological process					
GO:0008152	metabolic process	556	49	115	8.70E-16
GO:0006810	transport	450	39	68	0.0015
GO:0006629	lipid metabolic process	103	9	21	0.022
GO:0006548	histidine catabolic process	9	1	5	0.036
GO:0019441	tryptophan catabolic process to kynurenine	3	0	3	0.046
cellular component					
GO:0005777	peroxisome	20	2	8	0.016
molecular function					
GO:0003824	catalytic activity	648	57	124	1.50E-14
GO:0030170	pyridoxal phosphate binding	49	4	18	1.90E-05
GO:0016491	oxidoreductase activity	305	27	54	0.00012
GO:0005215	transporter activity	297	26	49	0.0026
GO:0004497	monooxygenase activity	77	7	19	0.004
GO:0009055	electron carrier activity	169	15	31	0.0097
GO:0020037	heme binding	117	10	24	0.0097
GO:0042626	ATPase activity, coupled to transmembrane movement of substances	30	3	10	0.016
GO:0005506	iron ion binding	138	12	26	0.018
GO:0004364	glutathione transferase activity	8	1	5	0.021
GO:0016712	oxidoreductase activity, acting on paired donors, with incorporation or reduction of molecular oxygen, reduced flavin or flavoprotein as one donor, and incorporation of one atom of oxygen	21	2	8	0.021
GO:0004252	serine-type endopeptidase activity	123	11	23	0.038
GO:0016769	transferase activity, transferring nitrogenous groups	14	1	6	0.048
GO:0016847	1-aminocyclopropane-1-carboxylate synthase activity	6	1	4	0.049

Appendix s11. Gene ontology terms that are overrepresented in genes that are primarily expressed in muscle. Terms with significant effects (Fisher's adjusted $p < 0.05$) are reported.

GO	go_description	total	expectation	observation	p
biological process					
GO:0006096	glycolysis	32	1	9	0.0038
GO:0008152	metabolic process	556	26	47	0.011
GO:0006122	mitochondrial electron transport, ubiquinol to cytochrome c	7	0	4	0.016
cellular component					
GO:0005739	mitochondrion	382	18	63	7.60E-16
GO:0005743	mitochondrial inner membrane	134	6	31	3.60E-11
GO:0030016	myofibril	5	0	4	0.0048
GO:0005747	mitochondrial respiratory chain complex I	4	0	3	0.03
molecular function					
GO:0004129	cytochrome-c oxidase activity	14	1	6	0.0048
GO:0008137	NADH dehydrogenase (ubiquinone) activity	16	1	6	0.0096
GO:0003824	catalytic activity	648	30	52	0.014
GO:0016616	oxidoreductase activity, acting on the CH-OH group of donors, NAD or NADP as acceptor	27	1	7	0.018
GO:0008121	ubiquinol-cytochrome-c reductase activity	8	0	4	0.023

Appendix s12. Gene ontology terms that are overrepresented in genes that are primarily expressed in skin. Terms with significant effects (Fisher's adjusted $p < 0.05$) are reported.

GO	go_description	total	expectation	observation	p
biological process					
GO:0008285	negative regulation of cell proliferation	82	5	18	0.00045
GO:0018149	peptide cross-linking	15	1	7	0.0023
GO:0050819	negative regulation of coagulation	9	1	5	0.014
GO:0007266	Rho protein signal transduction	11	1	5	0.037
cellular component					
GO:0005882	intermediate filament	82	5	25	4.00E-09
molecular function					
GO:0005200	structural constituent of cytoskeleton	93	6	28	6.00E-10
GO:0005544	calcium-dependent phospholipid binding	17	1	9	7.10E-05
GO:0004859	phospholipase inhibitor activity	10	1	6	0.0018
GO:0008092	cytoskeletal protein binding	37	2	9	0.037
GO:0005529	sugar binding	46	3	10	0.041

Appendix s13 . Gene ontology terms that are overrepresented in genes that are primarily expressed in spleen. Terms with significant effects (Fisher's adjusted p < 0.05) are reported.

GO	go_description	total	expectation	observation	p
biological process					
GO:0006412	translation	206	13	46	7.60E-12
GO:0007159	leukocyte adhesion	6	0	6	1.60E-05
GO:0006955	immune response	104	6	23	1.80E-05
GO:0007166	cell surface receptor linked signal transduction	38	2	10	0.012
cellular component					
GO:0005840	ribosome	148	9	43	2.70E-15
GO:0022627	cytosolic small ribosomal subunit	11	1	8	1.00E-05
GO:0009897	external side of plasma membrane	58	4	12	0.027
GO:0015935	small ribosomal subunit	11	1	5	0.039
molecular function					
GO:0003735	structural constituent of ribosome	140	9	43	5.10E-16
GO:0008009	chemokine activity	18	1	7	0.012
GO:0001948	glycoprotein binding	5	0	4	0.012

Appendix s14 . Gene ontolgy terms that are overrp resented ing enes that arep rimarily ep ressed in testes. Terms with significant effects (Fisher's adjusted p < 0.05) are reported.

GO	go_description	total	expectation	observation	p
biological process					
GO:0006886	intracellular protein transport	254	43	76	6.50E-05
GO:0007018	microtubule-based movement	76	13	30	0.00061
GO:0015031	protein transport	193	33	58	0.0011
GO:0007283	spermatogenesis	47	8	21	0.0013
GO:0043687	post-translational protein modification	37	6	17	0.0038
GO:0006139	nucleobase, nucleoside, nucleotide and nucleic acid metabolic process	41	7	17	0.013
GO:0051246	regulation of protein metabolic process	38	6	16	0.016
GO:0006511	ubiquitin-dependent protein catabolic process	109	18	34	0.018
GO:0000209	protein polyubiquitination	13	2	8	0.024
GO:0006465	signal peptide processing	8	1	6	0.028
GO:0000226	microtubule cytoskeleton organization	26	4	12	0.028
cellular component					
GO:0005875	microtubule associated complex	52	9	22	0.0019
GO:0005829	cytosol	149	25	45	0.0059
GO:0005874	microtubule	33	6	15	0.0099
GO:0005643	nuclear pore	30	5	13	0.034
GO:0005881	cytoplasmic microtubule	4	1	4	0.042
GO:0032391	photoreceptor connecting cilium	4	1	4	0.042
molecular function					
GO:0005515	protein binding	3375	569	666	8.20E-05
GO:0003777	microtubule motor activity	65	11	28	0.00015
GO:0003899	DNA-directed RNA polymerase activity	29	5	15	0.0023
GO:0031072	heat shock protein binding	50	8	21	0.003
GO:0008565	protein transporter activity	47	8	20	0.0035
GO:0019787	small conjugating protein ligase activity	37	6	17	0.0038
GO:0004842	ubiquitin-protein ligase activity	62	10	23	0.0099
GO:0000166	nucleotide binding	303	51	77	0.01
GO:0017111	nucleoside-triphosphatase activity	207	35	56	0.017
GO:0051082	unfolded protein binding	62	10	22	0.028
GO:0003924	GTPase activity	151	25	42	0.036
