# Supplementary material for: Digital gene expression analysis of the zebra finch genome
Source: BMC Genomics. 2010 Apr 1;11:219. doi: 10.1186/1471-2164-11-219 (PMC2996964; doi:10.1186/1471-2164-11-219)
Supplement: Additional file 2 — Appendix s15. Detailed survey of MHC genes [file 1471-2164-11-219-S2.PDF]

Appendix s15. Detailed presentation of the expression profiling in specific MHC genes.

Polymorphism of the major histocompatibility complex

Among the major histocompatibility complex (MHC) are some of the most widely used candidate genes in genomic studies. These highly variable loci are important for a number of ecologically relevant traits and processes. Most studies of avian MHC genes have, however, been limited to only one or few loci and to indirect genotyping approaches, such as RFLP and DGGE, where little is known about the loci being genotyped. Because of gene duplication and nonspecific PCR amplifications, locus-specific effects are often not investigated [1]. Typically studies of MHC variation have also focused on sequence variation only in a few highly polymorphic regions of class I and class II genes and variation in other genes, regions and expression levels has largely been ignored. To generate a broader picture of avian MHC genes, including their expression pattern, we thus choose to focus on these as a case study for the finding of candidate genes in transcriptomic next generation sequencing data.

Bird MHC organisation

The chicken MHC has been found to reside on a very compact genetic region on micro-chromosome 16. This region is made up of only approximately 40 genes, most of which are thought to play a role in adaptive immunity. In comparison to the mammal MHC, which consists of several hundred genes [2] this was a surprising finding and the term "minimal essential MHC" was coined to describe the chicken MHC [3]. Soon after the release of the chicken MHC sequence, there were speculations that the minimal essential MHC of the chicken was not very representative for birds in general [4, 5]. Many studies found a large number of gene duplications in the MHC genes of a wide variety of bird species [6, 7]. With the sequencing of the zebra finch MHC (Balakrishnan et al. in review) it is now clear that there are striking differences between the organisation and number of MHC genes between birds. The functionality of these differences can only be studied by taking patterns of gene expression into consideration.

Zebra finch MHC genes

Homologs to sixteen chicken MHC genes have been found in the zebra finch genome assembly. These include several class I and class II loci, but also other genes linked to the chicken MHC region (such as CD1, Blec and NKr) as well as genes situated outside the MHC region but with a function closely linked to MHC genes (such as B2M, Ii and CIITA). Some

additional loci have also been found using targeted sequencing of MHC containing zebra finch BACs (Balakrishnan et al. in review). Judging from the sequence data (stop codons and frame shift mutations), there seem to be several pseudogenes among the zebra finch MHC class I and class II genes.

Expression of MHC genes

Most of what is known about expression of MHC genes comes from studies of mammals [see for example 8], with only a few studies addressing MHC expression patterns in birds [for example 9, 10]. Expression of MHC class II genes is generally thought to be limited to antigen presenting cells of the immune system, while class I molecules are found on most nucleated cells in the body [11].

Methods for analysis of MHC gene expression

Coding sequences for all known zebra finch MHC genes (Balakrishnan et al. in review) were blasted (BLASTN) against the contigs from the full 454 data assembly, together with all singletons (reads that could not be joined to any contig during the assembly) and brain ESTs. For contigs, reads and ESTs giving significant hits ($e < 0.001$; a slightly less stringent cut-off e value used compared to that for the whole-genome survey because of the polymorphic nature of many MHC genes) against more than one MHC gene (due to recent duplications of several of the genes), all but the best (lowest e-value) were removed. All the sequences (contigs, singletons and ESTs) with significant hits against an MHC gene were then blasted (reciprocal BLASTN) against the full list of predicted zebra finch genes (including the manually annotated MHC genes), as well as the database of chicken proteins (BLASTX). The best hit for each query sequence was then extracted and manually checked for a good hit (reciprocal hit being the same as the original search gene). The number of transcripts for each MHC gene was then calculated for each tissue by counting the number of reads in each matching contig for the tissue in question and adding the number of singleton matches for the same tissue.

The assembly using zebra finch chromosome 16 and MHC containing BAC sequences as template were searched for regions with overlapping 454 reads or brain ESTs. All such regions were blasted against the NCBI nucleotide database, chicken reference protein dataset, zebra finch reference protein dataset and zebra finch genome assembly (using the NCBI online BLAST interface). Most (143 out of 232) of the region sequences made multiple strong

hits (e values approaching 0) to reference proteins and zebra finch genome regions, suggesting that these correspond to highly duplicated genes (such as zinc finger domains) or other highly repetitive genetic regions; these were excluded from further analyses. From the remaining 89 regions, 612 reads yielded unique hits to zebra finch and/or chicken genes, though not all of these were for MHC genes.

MHC class I and related

From the genome annotation there seem to be at least four MHC class I loci in the zebra finch but three of these have pseudogene characteristics (Appendix 16; Balakrishnan et al. in prep.). We found evidence for expression of one MHC class I locus (locus 1; Supplementary figure 1; Appendix 16). Expression of this gene is found in all sampled tissues but with the highest expression levels in spleen, brain and liver (Table 3). B2M (on chromosome 10), coding for the less variable part of the MHC class I molecule, shows a very similar pattern of expression but the levels are slightly lower than in the class I heavy chain (Table 3; Supplementary Figure 2). Of the transporters involved in MHC class I processing, only TAP1 has been found in the zebra finch genome assembly. TAP2 and TAP binding protein (tapasin) have instead been characterised from BAC sequencing (Balakrishnan et al. in prep.). We found no evidence for expression of TAP1 or 2 in the tissues analysed here. Four spleen reads and one from embryo assembled to the tapasin region of the zebra finch MHC-containing BACs, but these did not produce significant BLAST hits when screening them against the zebra finch or the chicken protein databases, suggesting that they may represent repetitive sequences in the genome. Sequences from all tissues except brain assembled onto an MHC-containing BAC region identified as the zebra finch gene "similar to class I MHC restricted T cell associated molecule" (CRTAM), suggesting that this gene may be linked to the zebra finch MHC region and that it is expressed at low to moderate levels in most tissues. No homologues to DMA or DMB have so far been found in the zebra finch genome or MHC-containing BACs.

MHC class II and related

We did not find evidence for expression of any of the nine MHC class IIB loci described in the zebra finch genome. The MHC class IIA gene is only partially described in the genome and it did not seem to appear in any of the sampled tissues either. In contrast, there were generally high levels of expression present in all investigated tissues (especially in spleen) for class II invariant chain (Ii, CD74; Supplementary Figure 3-4). This gene also seems to be the most evenly expressed of the MHC genes investigated here ($\tau = 0.336$; Table 3). Like the

chicken homologue, the Ii gene is found on chromosome 13 and there is evidence for expression of at least two alternatively spliced isoforms (Supplementary Figure 3). CIITA (chromosome 14) is the main transcription factor involved in class II regulation. It seems to be weakly expressed in zebra finch spleen (Table 3). BRD2 is a transcriptional factor mapping to the chicken MHC class II region, but it is not clear whether it is involved in regulation of immune genes. We found evidence for BRD2 expression in the brain EST libraries of zebra finch but not in the tissues analysed using 454 transcriptome sequencing (Table 3).

TRIM

In the chicken and other birds there are several tripartite motif (TRIM) genes in the extended MHC region. The TRIM genes are a very large family with a wide variety of functions in gene regulation and other cellular processes. Due to their highly duplicated nature it may be hard to establish orthologous relationships between pairs of genes from different species. At least four of the chicken MHC located TRIMs seem to have zebra finch homologues and of these we found evidence for expression of three. TRIM7.2 and TRIM27 seem to be primarily expressed in brain, whereas TRIM39 has low levels of expression in spleen, skin, testes and liver (Table 3).

CD1

Cluster of differentiation (CD) 1 proteins are involved in lipid antigen presentation to t-lymphocytes and are structurally and evolutionarily related to MHC genes [1, 2]. In chicken, two copies of CD1 genes are present just outside the MHC region on chromosome 16 [12]. In zebra finch the homologues to these are found on chromosome 12 of the assembly. We found evidence for expression of at least one of these genes with medium expression levels in spleen and low expression in liver (Table 3).

TUBB

Tubulin beta (TUBB) is involved in MHC class I function and cell mediated cytotoxicity. In mammals this gene maps to the MHC region but the location in chicken is unknown. In zebra finch, parts of a TUBB gene have been found, located on one of the MHC-containing BACs. We found low expression of this gene in brain and embryo (Table 3).

LAO

L-amino-acid oxidase precursor (LAO) is located among the TRIM genes of the chicken MHC region. Reads from a number of tissues (embryo, liver, muscle, skin and spleen) were assembled to a region on one of the MHC-containing BACs that came up as a LAO homologue when blasting against the chicken protein database.

KIFC1

Kinesin family member C1 (KIFC1) is located in the mammalian and chicken MHC region but it is not clear whether it is directly involved in immune function. In zebra finch the KIFC1 gene has been found on one of the MHC containing BACs but not in the genome assembly. Several reads from testes assembled onto this BAC region in our templated assembly. These reads did not, however, produce significant hits when blasting them back against the zebra finch or the chicken protein databases, suggesting that they may represent repetitive sequences in the genome.

FLOT1

Flotilin 1 (FLOT1) is located in mammalian MHC region and seems to be associated with neurological disease. It is not known, however, if it is directly involved in immune function. This gene has yet to be characterised in chicken. In zebra finch, FLOT1 has been found on one of the MHC-containing BACs. We found that several 454 reads from all investigated tissues assembled onto this BAC region. However, as for KIFC1, these sequences may represent repetitive sequences in the genome since they failed to produce good reciprocal BLAST hits.

Discussion

Traces of four different MHC class I loci have been found in the zebra finch genome, three of which were judged to represent pseudogenes due to frame shift mutations or premature stop codons (Balakrishnan et al. in review). The present study supports the conclusion that there is only one expressed MHC class I gene in the zebra finch, as the loci presumed to be pseudo genes (due to non-functional nucleotide sequences) do not seem to be expressed. Since MHC class I molecules are present on most nucleated cells it is not surprising that we found expression of this locus in all our sampled tissues, including brain. Class I expression in neurons is also known from studies of mammals [13, 14]. The zebra finch thus seem to be similar to chicken in the sense that it has only one predominantly expressed MHC class I

locus. This is in stark contrast to another passerine bird, the great reed warbler (*Acrocephalus arundinaceus*), that has at least four expressed MHC class I loci [15] , and provides tentative evidence that the structure of this gene is not uniform across passerines.

In contrast to class I genes, MHC class II loci are only expressed on specialised immune cells. Thus, our prediction was that we would only find evidence of class II expression in spleen and possibly liver. We did, however, not find any evidence for expression of MHC class II genes even in these tissues. It may be that these genes are expressed at levels too low for detection using our methodology, since only about 65% of the trascriptome was covered by our 454 generated ESTs. We did however find evidence of expression (again mainly in spleen) of the genes coding for class II invariant chain (Ii, CD74) and class II transactivator (CIITA), both of which are tightly linked to MHC class II function. Expression of several avian MHC genes (and other immune related genes) has also been shown to be dependent on infection status [10]. The individuals chosen for sequencing in this study appeared to be healthy, but a formal screen of infection status was not performed. It may be more likely to find expression of certain immune genes (such as MHC class IIB) in birds with an ongoing infection, or birds that have had their immune system artificially boosted by a vaccination prior to sampling.

Our study presents data from several different MHC genes and is not only focused on class I and class IIB genes as has often been the case in the past. The use of RNA-Seq to investigate expression profiles also represents a new direction in avian MHC studies. The results on the expression of MHC genes in various zebra finch tissues can be compared to expression of homologous genes in other organisms [16](www.hugeindex.org). Similar to our results, the human B2M is expressed in a wide variety of tissues but with a maximal expression in spleen. Also the human MHC class I (HLA-A) has a broad tissue expression with the highest levels in testes, blood and spleen. Of the human MHC class II genes, the HLA-DRB1 locus is the most highly expressed locus, with expression present in all tissues and with the highest value again in spleen. The human CIITA gene has very low expression levels and again, as in our study, spleen is the tissue of primary expression. High levels of expression of MHC, and other immune-related genes in spleen, have also been found in studies of fish [17] and other birds [4, 18-20]. Correlation of tissue expression signatures between different organisms seems to be a general pattern for many genes, and is probably due to evolutionary constraints on expression [21].

Supplementary Figures:

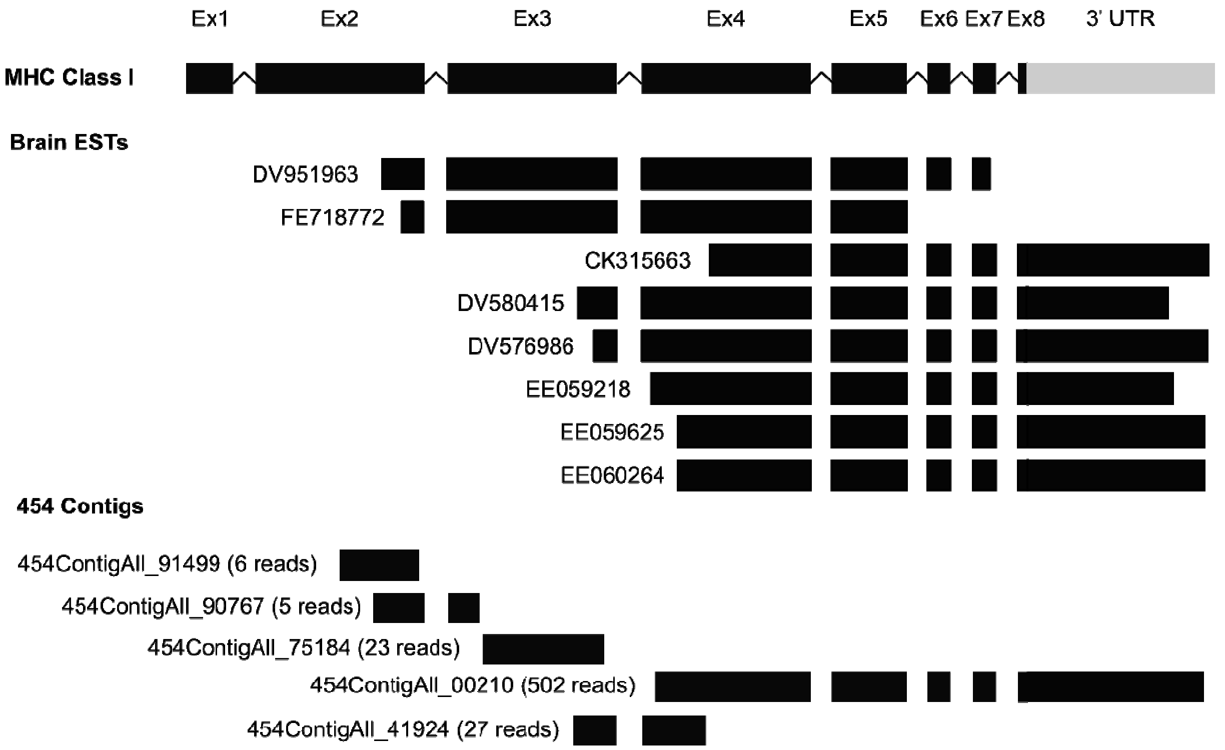

Supplementary Figure s1. Schematic alignment of 454 Contigs and Brain ESTs mapping to the zebra finch MHC class I gene. Note that although some of the 454 contigs overlap, the assembly program has failed to merge these into a single contig. This is likely because of the extreme polymorphism exhibited in certain regions of this gene. A sequence alignment of the zebra finch coding sequence for this gene and the 454 contigs represented here is given in Appendix 16.

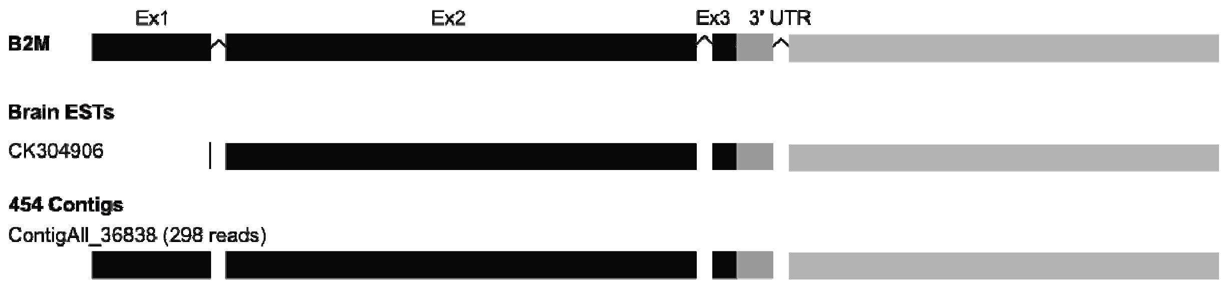

Supplementary figure s2. Schematic alignment of 454 Contigs and Brain ESTs mapping to the zebra finch B2M gene. For this relatively invariable gene, all 454 reads have been assembled into a single contig, covering most of the coding region and the 3'UTR.

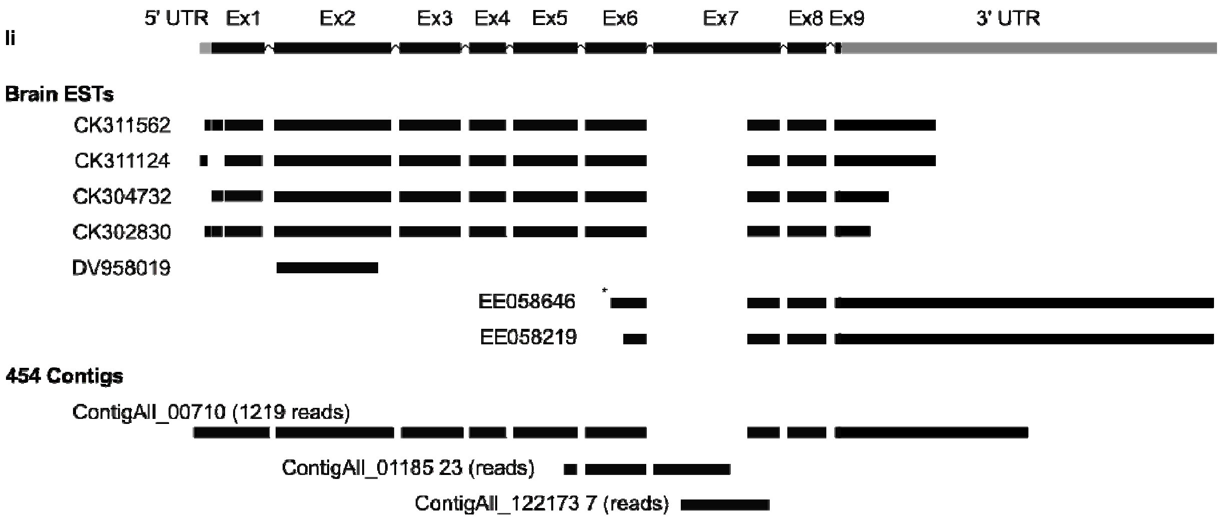

Supplementary Figure s3. Schematic alignment of 454 Contigs and Brain ESTs mapping to the zebra finch CD74 (Ii) gene. Note that at least two alternatively spliced isoforms are present in the 454 sequencing data and that these have been placed in different contigs during the assembly. A graph showing the sequence coverage of ContigAll_00710 is shown in Supplementary Figure 4.

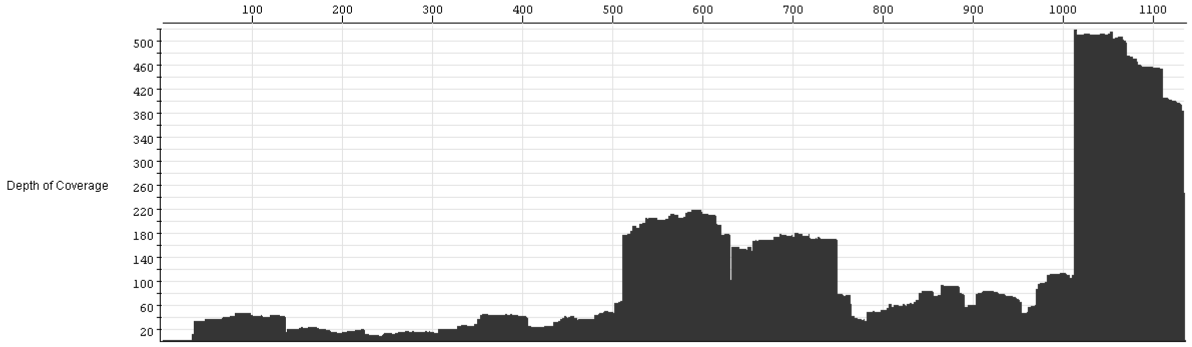

Supplementary Figure s4. Depth of sequence coverage (number of reads) for ContigAll_00710 from the assembly of the 454 sequencing data. This contig represents the most common isoform of the zebra finch CD74 (Ii) gene (see Supplementary Figure 3). The X-axis represents base pairs from the 5’ to the 3’ end of the contig. Notice the over-representation of reads in the right part of the figure representing the 3’un-translated region (UTR) of the gene.

References

1. Zelano B, Edwards SV: **An Mhc component to kin recognition and mate choice in birds: predictions, progress and prospects**. *Am Nat* 2002, **160**:S225-S237.
2. The MHC Sequencing Consortium: **Complete sequence and gene map of a human major histocompatibility complex**. *Nature* 1999, **401**:921-923.
3. Kaufman J, Milne S, Göbel TWF, Walker BA, Jacob JP, Auffray C, Zoorob R, Beck S: **The chicken B locus is a minimal essential major histocompatibility complex**. *Nature* 1999, **401**:923-925.
4. Shiina T, Shimizu S, Hosomichi K, Kohara S, Watanabe S, Hanzawa K, Beck S, Kulski JK, Inoko H: **Comparative genomic snalysis of two avian (Quail and Chicken) MHC regions**. *J Immunol* 2004, **172**(11):6751-6763.
5. Westerdahl H, Wittzell H, von Schantz T: **Mhc diversity in two passerine birds: no evidence for a minimal essential Mhc**. *Immunogenetics* 2000, **52**:92-100.
6. Westerdahl H: **Passerine MHC: genetic variation and disease resistance in the wild**. *Journal of Ornithology* 2007, **148**:S469-S477.
7. Ekblom R, Grahn M, Hoglund J: **Patterns of polymorphism in the MHC class II of a non-passerine bird, the great snipe (Gallinago media)**. *Immunogenetics* 2003, **54**(10):734-741.
8. Ting JP-Y, Baldwin AS: **Regulation of MHC gene expression**. *Current Opinion in Immunology* 1993, **5**(1):8-16.
9. Kaufman J, Salomonsen J: **The "minimal essential MHC" revisited: Both peptide-binding and cell surface expression level of MHC molecules are polymorphisms selected by pathogens in chickens**. *Hereditas* 1997, **127**:67-73 1997.
10. Wang Z, Farmer K, Hill GE, Edwards SV: **A cDNA macroarray approach to parasite-induced gene expression changes in a songbird host: genetic response of house finches to experimental infection by *Mycoplasma gallisepticum***. *Molecular Ecology* 2006, **15**(5):1263-1273.
11. Roitt IM: **Essential immunology**, 9th ed. edn. Oxford: Blackwell Science Ltd; 1997.
12. Salomonsen J, Sørensen MR, Marston DA, Rogers SL, Collen T, van Hateren A, Smith AL, Beal RK, Skjødt K, Kaufman J: **Two CD1 genes map to the chicken MHC, indicating that CD1 genes are ancient and likely to have been present in the primordial MHC**. *Proceedings of the National Academy of Sciences of the United States of America* 2005, **102**(24):8668-8673.
13. Boulanger LM, Shatz CJ: **Immune signalling in neural development, synaptic plasticity and disease**. *Nat Rev Neurosci* 2004, **5**(7):521-531.
14. Huh GS, Boulanger LM, Du H, Riquelme PA, Brotz TM, Shatz CJ: **Functional requirement for class I MHC in cns development and plasticity**. *Science* 2000, **290**(5499):2155-2159.
15. Westerdahl H, Wittzell H, von Schantz T: **Polymorphism and transcription of Mhc class I genes in a passerine bird, the great reed warbler**. *Immunogenetics* 1999, **49**:158-170.
16. Hsiao L-L, Dangond F, Yoshida T, Hong R, Jensen RV, Misra J, Dillon W, Lee KF, Clark KE, Haverty P *et al*: **A compendium of gene expression in normal human tissues**. *Physiol Genomics* 2001, **7**(2):97-104.
17. Kocabas AM, Li P, Cao D, Karsi A, He C, Patterson A, Ju Z, Dunham RA, Liu Z: **Expression profile of the channel catfish spleen: analysis of genes involved in immune functions**. *Marine Biotechnology* 2002, **4**(6):526-536.
18. Zoorob R, Béhar G, Kroemer G, Auffray C: **Organization of a functional chicken class II B gene**. *Immunogenetics* 1990, **31**:179-187.

19. Kaufman J, Jacob J, Shaw J, Walker B, Milne S, Beck S, Salomonsen J: **Gene organisation determines evolution of function in the chicken MHC**. *Immunological Reviews* 1999, **167**(1):101-117.
20. Strand T, Westerdahl H, Höglund J, V. Alatalo R, Siitari H: **The Mhc class II of the Black grouse (Tetrao tetrix) consists of low numbers of B and Y genes with variable diversity and expression**. *Immunogenetics* 2007, **59**(9):725-734.
21. Chan ET, Quon GT, Chua G, Babak T, Trochesset M, Zirngibl RA, Aubin J, Ratcliffe MJ, Wilde A, Brudno M *et al*: **Conservation of core gene expression in vertebrate tissues**. *Journal of Biology* 2009, **8**:33.
