# Supplementary material for: Digital gene expression analysis of the zebra finch genome
Source: BMC Genomics. 2010 Apr 1;11:219. doi: 10.1186/1471-2164-11-219 (PMC2996964; doi:10.1186/1471-2164-11-219)
Supplement: Additional file 3 — Appendix s16. Alignment of the zebra finch MHC class I gene [file 1471-2164-11-219-S3.PDF]

```
                                 10        20        30        40        50        60        70        80        90       100
                        ....|....|....|....|....|....|....|....|....|....|....|....|....|....|....|....|....|....|....|....|
Tg locus 1 cds          ATGGCAGGGCCGGAGGCGGCTCTGGGGGCGGGGCCGGGGCTGGGGCGGGGCCGGGGCGGTGCCCGGGGCCGCATTCTCCACTCCCTGCATTACCTGCACG
Tg pseudo L             ATGTGGGCTAGGGGGGGATGC-------TGAGGGCAGGACCAGGGGGTGACAGTGCCCAGCCTGGCTGGGGCTTTTTCCACTCCCTGCAGTTCCTGGAAG
Tg pseudo O             -------------------------------------------------------------------------TTCTCCACTCCCTGCAGTTCCTGGAAG
Tg pseudo C             ----------------------------------------------------------------------------------------------------
454ContigAll_91499      ----------------------------------------------------------------------------------------------------
454ContigAll_90767      ----------------------------------------------------------------------------------------------------
454ContigAll_75184      ----------------------------------------------------------------------------------------------------
454ContigAll_41924      ----------------------------------------------------------------------------------------------------
454ContigAll_00210      ----------------------------------------------------------------------------------------------------

                                110       120       130       140       150       160       170       180       190       200
                        ....|....|....|....|....|....|....|....|....|....|....|....|....|....|....|....|....|....|....|....|
Tg locus 1 cds          TGGCGGTGTCAGAGCCCAGCCCGGGGGTCCCCCAGTTCACGAGCATTGGGTTTGTGGATGGGATCCCCTTCGTGCGCTACGACAGCGAGCGGGGCCGGGC
Tg pseudo L             TGTTGCTGTCAGAGCCCGGCCCGGGGGTCCCCCAGTCCTTGATTGTGGGGGACGTGGACGGGACCCCCTGCGAGCGCTGCGGCAGCGAGCGGGGCCGGAT
Tg pseudo O             TGGTGCTATCAGAGCCCGGCCCGGGGGTCCCCCAGTTCTTGTTTGGGGGGGACGTGGACGGGACCCTCTGCGAGCGCTGCGGCAGCGAGCGGGGCCGGAT
Tg pseudo C             ----------------------------------------------------------------------------------------------------
454ContigAll_91499      --------------------------------------------------------------------------------------------------GT
454ContigAll_90767      ----------------------------------------------------------------------------------------------------
454ContigAll_75184      ----------------------------------------------------------------------------------------------------
454ContigAll_41924      ----------------------------------------------------------------------------------------------------
454ContigAll_00210      ----------------------------------------------------------------------------------------------------

                                210       220       230       240       250       260       270       280       290       300
                        ....|....|....|....|....|....|....|....|....|....|....|....|....|....|....|....|....|....|....|....|
Tg locus 1 cds          GGAGCCGCTGACACAGTGGATGAAGGATGGAGCCGAGCCGGGATATTGGGATGAGGAGACCCAGATTTCTGTGAGGAACCAGCACGTGGATGCCAGGAAC
Tg pseudo L             GGAGGCACAGACACCAGGGATGGGGACGGGAGCTGAGCTGGGATATTGGGA-------------------------------------------------
Tg pseudo O             GGAGGCACAGACACCAGGGATGGGGGCGGGAGCTGAGCTGGGATATTGGGACAGCCA-------------------------------------------
Tg pseudo C             ----------------------------------------------------------------------------------------------------
454ContigAll_91499      GGAGCCGCTGACRCAGTGGATGAAGGATGGAGCCGAGCCGGGATATTGGGATGGGCAGACCCAGAGTGCTGTGAGGAACCAGCACGTGGCTGCCAGGAAC
454ContigAll_90767      --------------------------------------------------------AGACCCAGATTTCTGTGAGGAACCAGCACGTGGCTGCCAGGAGC
454ContigAll_75184      ----------------------------------------------------------------------------------------------------
454ContigAll_41924      ----------------------------------------------------------------------------------------------------
454ContigAll_00210      ----------------------------------------------------------------------------------------------------

                                310       320       330       340       350       360       370       380       390       400
                        ....|....|....|....|....|....|....|....|....|....|....|....|....|....|....|....|....|....|....|....|
Tg locus 1 cds          CTGGAGACACTGCAGGAGCGGTACAACCAGAGCAGGGGTCTCCACACTGCGTTGTGGGCTTATGGCTGTGACCTCCTGTCCGATGGGAGTGTCCGCGGAT
Tg pseudo L             -------------------------------------GTCTCCACACAGTGCAGGGGGTTTCCAGCTGTGACCTCCTGTCCAATGGAGCATCCATGGATC
Tg pseudo O             ----------------------------------------------------------------------------------------------------
Tg pseudo C             ----------------------------------------------------------------------------------------------------
454ContigAll_91499      CTGGAGATACTGCAGGAGCGGTACAACCAG----------------------------------------------------------------------
454ContigAll_90767      CTGGAGATACTGCAGGAGCGGTACAACCAGAGCAGGGGTCTCCACACTGTGTTGAGGGCTTATGGCTGTGACCTCCTGTCCG------------------
454ContigAll_75184      ----------------------------------------------------------------------------------------------------
454ContigAll_41924      ----------------------------------------------------------------------------------------------------
454ContigAll_00210      ----------------------------------------------------------------------------------------------------

                                410       420       430       440       450       460       470       480       490       500
                        ....|....|....|....|....|....|....|....|....|....|....|....|....|....|....|....|....|....|....|....|
Tg locus 1 cds          CCCGCCGGTATGGCTACGACGGGCGGGATTTCATCTCCTTTGACCTGGGCTCCGGGAAATTCCTGGCGGCCGACAGCGCTGCCGAGATCACCAGGAGGCG
Tg pseudo L             CCACCAGTACAGCTACGAGGGGCTGGGATTTCATCTCTTTCCTGCTGGGATCCAGGAGCTTTGCAGTGTCCAGCGGTGCCACCTGGATATCCCAGAGGTG
Tg pseudo O             ----------------------------------------------------------------------------------------------------
Tg pseudo C             ----------------------------------------------------------------------------------------------------
454ContigAll_91499      ----------------------------------------------------------------------------------------------------
454ContigAll_90767      ----------------------------------------------------------------------------------------------------
454ContigAll_75184      ---TCCGGGATGGCTACGACGGGCGGGATTTCATCTCCTTTGACCTGGGCTCCGGGAAATTCCTGGCGGCCGACAGCGCTGCCGAGATCACCAGGAGGCG
454ContigAll_41924      ----------------------------------------------------------------------------------------------------
454ContigAll_00210      ----------------------------------------------------------------------------------------------------

                                510       520       530       540       550       560       570       580       590       600
                        ....|....|....|....|....|....|....|....|....|....|....|....|....|....|....|....|....|....|....|....|
Tg locus 1 cds          CTGGGAG---CAGGAGGACATGGCTGAGAGGCTTAAAAATTACCTGAAGCACAAATGCCCTGAATGGCTCCGGAAATACGTTGGATACGGGCAGAAGGAG
Tg pseudo L             CTGGGAACACAAAGGGATCATGGTGGAGCAGATGAAGCATTACCTGGGCCACACCTGTGTGGAAAGGCCCCCAAAATACATCAGATATGGTCGGGAGGCT
Tg pseudo O             ----------------------------------------------------------------------------------------------------
Tg pseudo C             ----------------------------------------------------------------------------------------------------
454ContigAll_91499      ----------------------------------------------------------------------------------------------------
454ContigAll_90767      ----------------------------------------------------------------------------------------------------
454ContigAll_75184      CTGGGAG---CAGGAGGAGGAGGCTGAGAGGTGGACATATTACCTGAAGCACGAATGCCCTGAATGGCTCCGGAAATACGTTGGATACGGGCAGAAGGA-
454ContigAll_41924      ------------------------------------------------------------TGAATGGCTCCGGAAATACGTTGGATACGGGCAGAAGGAG
454ContigAll_00210      ----------------------------------------------------------------------------------------------------

                                610       620       630       640       650       660       670       680       690       700
                        ....|....|....|....|....|....|....|....|....|....|....|....|....|....|....|....|....|....|....|....|
Tg locus 1 cds          CTGGAGCGCAAAGAGCCCCCTGATGTCCACGTGTCCGGAAAAGAGGAATACGGGACGCTGATCCTGTCCTGCCACGCATACGGATTCTACCCCAACACCA
Tg pseudo L             CTGGAGCACAAAG---------------------------------------------------------------------------------------
Tg pseudo O             ----------------------------------------------------------------------------------------------------
Tg pseudo C             -------------AACCCCCTGATGCTCATGTGTACAGAAAAGTGGAACATGGGACCCGGGCCCTGTCCTGCCACATGTATGGATTCTGCCCCAGGACCA
454ContigAll_91499      ----------------------------------------------------------------------------------------------------
454ContigAll_90767      ----------------------------------------------------------------------------------------------------
454ContigAll_75184      ----------------------------------------------------------------------------------------------------
454ContigAll_41924      CTGGAGCGCAAAGAGCCCCCTGATGTCCACGTGTCCGGAAAAGAGGAATACGGGACGCTGATCCTGTCCTGCCACGCGTACGGATTCTACCCAAAAACAA
454ContigAll_00210      ------------------------------------ACGCATCCGACGCGGGGGACATTGATCCTGTCCTGCCACGCGTACGGATTCTACCCCAACACCA
```

```
                                710       720       730       740       750       760       770       780       790       800
                       ....|....|....|....|....|....|....|....|....|....|....|....|....|....|....|....|....|....|....|....|
Tg locus 1 cds         TCACAGTCAGCTGGATGAAGGAGAATGCAACCTTGGATCAGGAGATGGAGTGGGGCGGGATCGTTCCCAACAGCGATGGCACCTTCCACACCTGGGCCAG
Tg pseudo L            ----------------------------------------------------------------------------------------------------
Tg pseudo O            ----------------------------------------------------------------------------------------------------
Tg pseudo C            TCAGGATGAGCTGGATGAAGGGGGAGGAAATGCAGGATCAGGAGATTGAGTTGGGTGGAATCATTCCCAACAGCAATGGCACCTTCCACACCTGGGCCAG
454ContigAll_91499     ----------------------------------------------------------------------------------------------------
454ContigAll_90767     ----------------------------------------------------------------------------------------------------
454ContigAll_75184     ----------------------------------------------------------------------------------------------------
454ContigAll_41924     CCAATACAACAAGTACAAG---------------------------------------------------------------------------------
454ContigAll_00210     TCACAGTCAGCTGGATGAAGGAGAATGCAACCTTGGATCAGGAGATGGAGTGGGGCGGGATCGTTCCCAACAGCGATGGCACCTTCCACACCTGGGCCAG

                                810       820       830       840       850       860       870       880       890       900
                       ....|....|....|....|....|....|....|....|....|....|....|....|....|....|....|....|....|....|....|
Tg locus 1 cds         GATCGAGGCGCTGCCGGAGGAGTGGGAGCAGTACCGGTGCAAGGTGGACCATCCCGGAATGCTGGAGCCCGGGCTCTTCGCTTGGGAGCCGACGTCTGGC
Tg pseudo L            ----------------------------------------------------------------------------------------------------
Tg pseudo O            ----------------------------------------------------------------------------------------------------
Tg pseudo C            AGCTGAGGCACTGCCAGGGGAGCGGGAGCAGCACTGGTGCCGGGTGGAGCATCCCAGGATGCTGGAGCTTGGGATCCTCATCTGGGAGCTGGAATCAAGC
454ContigAll_91499     ----------------------------------------------------------------------------------------------------
454ContigAll_90767     ----------------------------------------------------------------------------------------------------
454ContigAll_75184     ----------------------------------------------------------------------------------------------------
454ContigAll_41924     ----------------------------------------------------------------------------------------------------
454ContigAll_00210     GATCGAGGCGCTGCCGGAGGAGTGGGAGCAGTACCGGTGCAAGGTGGACCATCCCGGAATGCTGGAGCCCGGGCTCTTCGCTTGGGAGCCGACGTCTGGC

                                910       920       930       940       950       960       970       980       990      1000
                       ....|....|....|....|....|....|....|....|....|....|....|....|....|....|....|....|....|....|....|
Tg locus 1 cds         AGGAATCTCACCATGGCGGTTGCTGTGTCTGTCATCGCTGCCATCCTCATCCTCACTGTCCTCATCGGATTCGGTGTCTGGAAGCTCCAATCCGGGAGGA
Tg pseudo L            ----------------------------------------------------------------------------------------------------
Tg pseudo O            ----------------------------------------------------------------------------------------------------
Tg pseudo C            TGGAATCTCATCCTGGTGATGGTCGCTGTGTCTGTCATTGCTATG-TCATGATCCTCATTGGATTCAGGATCTGGAAGGTCCGACCTGGTAATGGAAAAG
454ContigAll_91499     ----------------------------------------------------------------------------------------------------
454ContigAll_90767     ----------------------------------------------------------------------------------------------------
454ContigAll_75184     ----------------------------------------------------------------------------------------------------
454ContigAll_41924     ----------------------------------------------------------------------------------------------------
454ContigAll_00210     AGGAATCTCACCATGGCGGTTGCTGTGTCTGTCATCGCTGCCATCCTCTTCCTCACTGTCCTCATCGGATTCGGTGTCTGGAAGCTCCAATCTGGGAGGA

                               1010      1020      1030      1040      1050      1060      1070      1080
                       ....|....|....|....|....|....|....|....|....|....|....|....|....|....|....|....|...
Tg locus 1 cds         GGGACAGGAGTGGATACAACGTGGCAGCCGGGAAGGACGTGGGAATGAATGGCTCAACCGCAGGGATTCCAGTGCAGGAGTGA
Tg pseudo L            -----------------------------------------------------------------------------------
Tg pseudo O            -----------------------------------------------------------------------------------
Tg pseudo C            GGAGGGGAATGGACACAGTACGGTGGTCA------------------------------------------------------
454ContigAll_91499     -----------------------------------------------------------------------------------
454ContigAll_90767     -----------------------------------------------------------------------------------
454ContigAll_75184     -----------------------------------------------------------------------------------
454ContigAll_41924     -----------------------------------------------------------------------------------
454ContigAll_00210     GGGACAGGAGTGGATACAACGTGGCAGCCGGGAAGGACGTGGGAATGAATGGCTCAACCGCAGGAATCACCGCCTGAGCGATC
```

Appendix 16. Sequence alignment of coding sequences (cds) from Zebra finch MHC class I loci (one functional loci and three putative pseudo-genes; see Balakrishnan et al. in review) and 454 sequencing contigs matching these in the present dataset. Similarity to the top sequence (expressed loci) is indicated with a solid outline. Note that all the 454 contigs are more similar to the expressed loci than any of the putative pseudo-genes.
